# Chromium toxicity, speciation, and remediation strategies in soil-plant interface: A critical review

**DOI:** 10.3389/fpls.2022.1081624

**Published:** 2023-01-13

**Authors:** Usman Zulfiqar, Fasih Ullah Haider, Muhammad Ahmad, Saddam Hussain, Muhammad Faisal Maqsood, Muhammad Ishfaq, Babar Shahzad, Muhammad Mohsin Waqas, Basharat Ali, Muhammad Noaman Tayyab, Syed Amjad Ahmad, Ilyas Khan, Sayed M. Eldin

**Affiliations:** ^1^ Department of Agronomy, Faculty of Agriculture and Environment, The Islamia University of Bahawalpur, Bahawalpur, Pakistan; ^2^ Key Laboratory of Vegetation Restoration and Management of Degraded Ecosystems, South China Botanical Garden, Chinese Academy of Sciences, Guangzhou, China; ^3^ Center of Plant Ecology, Core Botanical Gardens, Chinese Academy of Sciences, Guangzhou, China; ^4^ Department of Agronomy, University of Agriculture, Faisalabad, Pakistan; ^5^ Department of Botany, The Islamia University of Bahawalpur, Bahawalpur, Pakistan; ^6^ Tasmanian Institute of Agriculture, University of Tasmania, Hobart, TAS, Australia; ^7^ Department of Agricultural Engineering, Khwaja Fareed University of Engineering and Information Technology (KFUEIT), Rahim Yar Khan, Pakistan; ^8^ Department of Plant Breeding and Genetics, Ghazi University, Dera Ghazi Khan, Pakistan; ^9^ Department of Mechanical Engineering, NFC IEFR, Faisalabad, Pakistan; ^10^ Department of Mathematics, College of Science Al-Zulfi, Majmaah University, Al-Majmaah, Saudi Arabia; ^11^ Center of Research, Faculty of Engineering, Future University in Egypt, New Cairo, Egypt

**Keywords:** chromium phytotoxicity, environment, contamination, plant physiology and growth, remediation

## Abstract

In recent decades, environmental pollution with chromium (Cr) has gained significant attention. Although chromium (Cr) can exist in a variety of different oxidation states and is a polyvalent element, only trivalent chromium [Cr(III)] and hexavalent chromium [Cr(VI)] are found frequently in the natural environment. In the current review, we summarize the biogeochemical procedures that regulate Cr(VI) mobilization, accumulation, bioavailability, toxicity in soils, and probable risks to ecosystem are also highlighted. Plants growing in Cr(VI)-contaminated soils show reduced growth and development with lower agricultural production and quality. Furthermore, Cr(VI) exposure causes oxidative stress due to the production of free radicals which modifies plant morpho-physiological and biochemical processes at tissue and cellular levels. However, plants may develop extensive cellular and physiological defensive mechanisms in response to Cr(VI) toxicity to ensure their survival. To cope with Cr(VI) toxicity, plants either avoid absorbing Cr(VI) from the soil or turn on the detoxifying mechanism, which involves producing antioxidants (both enzymatic and non-enzymatic) for scavenging of reactive oxygen species (ROS). Moreover, this review also highlights recent knowledge of remediation approaches i.e., bioremediation/phytoremediation, or remediation by using microbes exogenous use of organic amendments (biochar, manure, and compost), and nano-remediation supplements, which significantly remediate Cr(VI)-contaminated soil/water and lessen possible health and environmental challenges. Future research needs and knowledge gaps are also covered. The review’s observations should aid in the development of creative and useful methods for limiting Cr(VI) bioavailability, toxicity and sustainably managing Cr(VI)-polluted soils/water, by clear understanding of mechanistic basis of Cr(VI) toxicity, signaling pathways, and tolerance mechanisms; hence reducing its hazards to the environment.

## 1 Introduction

Heavy metal contamination has disastrous impacts on terrestrial as well as aquatic life (Pushkar et al., 2021), and it has significantly disrupted the natural ecosystem (Zulfiqar et al., 2022). The unplanned urban and industrial development that disregards the value of a healthy environment is the main cause of environmental pollution (Dabir et al., 2019; Wei et al., 2022a). These actions have greatly increased the pollution from heavy metals, which upsets the natural balance (Posthuma et al., 2019; Qianqian et al., 2022). More than 1.7 million deaths were reported by World Health Organization (WHO) because of exposure to harmful contaminants, such as heavy metals (World Health Organization (WHO), 2017; Xu et al., 2018). The increase of heavy metal pollution in the environment increases the potential of human exposure to these heavy metals (Zulfiqar et al., 2019). Heavy metals may be harmful to living things due to their biodegradable properties (Qianqian et al., 2022). At different trophic levels, heavy metals frequently bioaccumulate and move within the ecosystem (Pushkar et al., 2021). Untreated trash can contain heavy metals that may leak into irrigation water/groundwater and easily absorbed by plants (Banerjee et al., 2019). Heavy metals can have fatal consequences on living things when they encounter them through water, air, food, etc. (Majumder et al., 2017; Yaashikaa et al., 2019). The degradation of heavy metals is a serious problem that requires immediate action.

**Figure 1:**
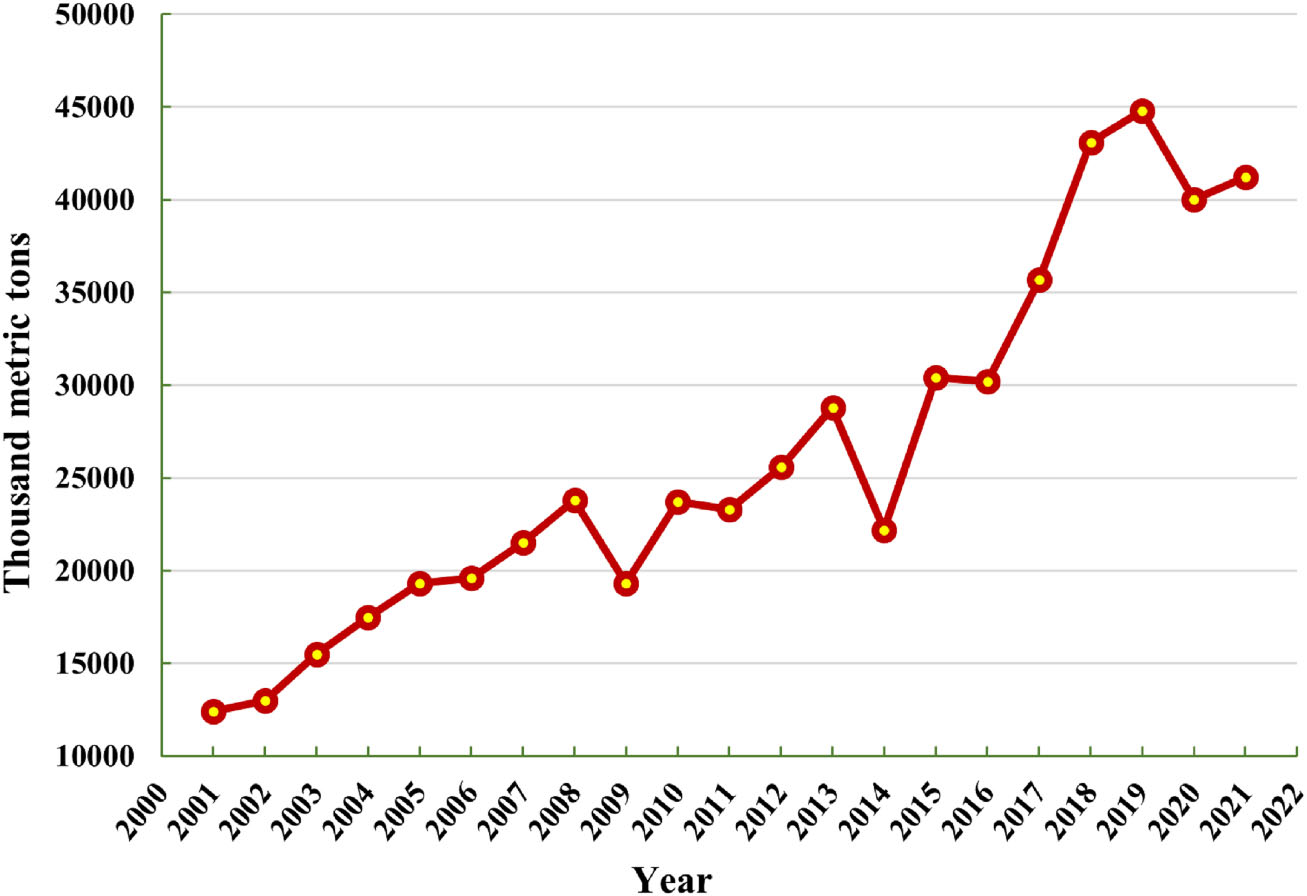
Annual world mine production of Cr in thousand metric tons (source, U.S.G.S. (United States Geological Survey), 2021).

In the earth’s mantle, chromium (Cr) is 17^th^ the most plenteous element, and the valence state of Cr regulates its toxicity in plants. Cr is widely used in a various industry, including the Cr plating, tanneries, mining, steel, and chemical industry (Shahid et al., 2017; Pushkar et al., 2021). Cr has become more prevalent as an environmental pollutant due to its increased industrial uses (Pradhan et al., 2019; Wei et al., 2022a). Cr is a pervasive contaminant with significant environmental hazards, particularly for soil-plant ecosystem (Ao et al., 2022; Kapoor et al., 2022). It is a metallic compound that belongs to category VI-B in the periodic table with an atomic number of 24. It is a shiny, hard, and steel-gray mineral with maximum melting point (Owlad et al., 2009). The annual world mine production of Cr in thousand metric tons is mentioned in **Figure 1**. The trivalent and hexavalent Cr appears being the most persistent among the numerous chromium oxidation states (III to +VI) (Chug et al., 2016). Hexavalent Cr is known to be a dangerous metal relative to the trivalent form because of its carcinogenic, mutagenic, and oxidizing properties (Wei et al., 2022a). Compounds of Cr(VI) are thousand times more cytostatic and carcinogenic than Cr(III) (Mamais et al., 2016). Furthermore, as opposed to further forms, Cr(VI) is highly soluble and bioavailable, obtaining more consideration (Xiao W. et al., 2017). There is no known biological function of Cr in plants (Srivastava et al., 2021). The soil properties, such as soil texture, pH, organic matter (OM) composition, electrical conductivity (EC), sulphide ions, iron (Fe) and manganese (Mn) oxides, microbial activity, and soil moisture content, as well as the plant physiology, such as root surface area, rate of root exudation, rate of transpiration, and plant type all influence the biogeochemical behavior of Cr in soil-plant systems (Shahid et al., 2017; Xiao et al., 2021; Ao et al., 2022). Plants lack specialized transporters and channels for absorbing Cr because it is a non-essential element for them (Adhikari et al., 2020). As a result, certain carriers of the necessary ions for plant metabolism, such as Fe for Cr(III) and phosphate and sulphate for Cr(VI), are used by plants to accumulate Cr (Anjum et al., 2016a). The oxidative stress caused due to Cr toxicity may lead to reduce membrane stability due to the over-accumulation of reactive oxygen species (ROS) that may also damage the morpho-physiological attributes in the plants (Eleftheriou et al., 2015; Azeez et al., 2021). Due to oxidative reactions such as mutilation of DNA and RNA, inhibition of enzymes, lipid peroxidation, and protein oxidation, ROS can induce cell death when produced in high concentrations (Srivastava et al., 2021). The functioning and regulation of many proteins are reportedly suppressed by Cr toxicity (Dotaniya et al., 2014; Handa et al., 2018a), and plant tissues exhibit chromosomal abnormalities as a result (Shahid et al., 2017). Numerous techniques, including solvent extraction, adsorption, chemical reduction, bio-remediation, and others, have been thoroughly investigated and evaluated, to remove hazardous form Cr(VI) to non-toxic Cr(III) form from polluted soil, water, and air (Azeez et al., 2021). Moreover, plants have evolved a variety of sophisticated adaptation methods, such as chelation by organic compounds followed by sequestration within vacuoles, to deal with high amounts of ROS produced under biotic and abiotic challenges (Azeez et al., 2021; Pushkar et al., 2021). To combat the elevated amounts of Cr-mediated ROS, plants also have a secondary mechanism for generating antioxidant enzymes (Srivastava et al., 2021; Ao et al., 2022). Understanding the biogeochemistry of Cr in soil-plant environments and the effects that high levels of Cr will have on the ecosystem is crucial.

The effects of Cr toxicity on agricultural productivity, lipid peroxidation, ROS production, and potential remediation procedures have been described in a number of previous research (Shanker et al., 2005; Shahid et al., 2017; Azeez et al., 2021; Srivastava et al., 2021; Ao et al., 2022). This review provides an overview of the most recent research on the mechanisms underlying transport of Cr, accumulation, toxicity, and detoxification in soil-plant systems. The toxic effects of Cr on key metabolic functions of plants leading to growth and yield impairment are reported. The mechanisms of Cr(VI) immobilization and reduction by organic amendments i.e., biochar, compost, and organic manure are also discussed Cr(VI). Additionally, in this review the recent remediation techniques are also highlighted, such as bioremediation, which includes phytoremediation, remediation using microbes, and supplements for nano-remediation. These techniques significantly reclaim Cr-contaminated soil and water while reducing potential health and environmental risks. To define future research goals and needs, research gaps in the biogeochemical behavior of Cr in soil-plant systems and difficulties in using *in-situ* remediation materials for Cr(VI)-contaminated soils are also integrated.

## 2 Chemical properties of chromium

The element Cr is relatively active. Instead of reacting with water, it reacts with many acids. At room temperature, it reacts with oxygen to create chromium oxide (Cr_2_O_3_). A thin layer of chromium oxide coats the metal’s surface, preventing further corrosion (rusting). The atomic number of Cr is 24 and has 51.996 g mol^-1^ molecular weight. Moreover, the electronegativity of Cr is 1.6, density 7.19 g cm^-3^ at 20°C, ionic radius 0.061 nm for Cr(III) and 0.044nm for Cr(VI), melting point 1907°C and boiling point is 2672°C. Cr is hard, brittle, and lustrous. It can be highly polished and is a silver-gray color.

## 3 Sources of chromium in environment

Cr is one of the heavy metals whose concentration is continuously rising because of industrial expansion and combustion processes, particularly the rise of the metal, chemical, and tanning sectors (Sharma et al., 2020). Industrial processes like leather tanning, Cr plating, pigment production, wood preservation, and the use of Cr as a corrosion-inhibitor in cooling towers are examples of anthropogenic sources (Shanker et al., 2005). Natural sources include the leaching of Cr during weathering of ultramafic rocks is another (Ao et al., 2022). Other environmental sources of Cr include power plants using liquid fuels, brown, and hard coal, industrial and municipal trash, and rocks eroded by water and air (Shahid et al., 2017). Cr pollution is not a concern on a global basis, but it may cause excessive concentrations of this pollutant to circulate in the biogeochemical cycle locally due to metal permeability into soil, water, or the atmosphere (Yang et al., 2022).

## 4 Chromium dynamics in soil

The average soil concentration of Cr is about 40 mg kg^-1^ (Isak et al., 2013). Cr exhibits a wide range of potential states of oxidation, the +3 state is vigorously persistent; the +3 and +6 forms are frequently seen in Cr groups, while the +1, +4, and +5 states are uncommon. Cr FeCr_2_O_4_ chromate, which contains about 70% of pure Cr_2_O_3_, is the main mineral possessing this element (Lakshmi and Sundaramoorthy, 2010). Natural Cr exists in most soils as relatively inert forms of Cr(III) that must be liberated over time by acid discharge (Chandra et al., 2010). The manganese (Mn) oxides present in soils will oxidize Cr(III) into Cr(VI), but a minute proportion of Cr(III) in soils is typically found in oxidizable forms (Mishra et al., 2009). Within the soil, Cr is perfectly integrated, however effectively bound to organic materials on Fe and Mn oxides and hydroxides (Balamurugan et al., 2014).

## 5 Factors affecting chromium dynamics

The disruption of the equilibrium state between species is significantly impacted by several chemical events that Cr conversion can cause in soils, including hydrolysis, oxidation, precipitation, and reduction (Di-Palma et al., 2015). Shift of redox state (Eh), soil pH, cation exchange capacity (CEC), biological conditions, microbial environments, and competitive cations have a significant impact on these complex interactions (Taghipour and Jalali, 2016). Cr speciation is particularly vulnerable to the values of soil Eh (Xiao et al., 2019). The dominant factor influencing soil Eh may be biochemical properties of metals, specifically those with different types of metal oxidation conditions in the soil (Bell et al., 2022). In addition to Cr(III) immobilization and precipitation, altered soil types cause hazardous Cr(VI) to be converted to less harmful Cr(III) (Pakade et al., 2019). Generally, in oxygen-rich conditions, Cr(VI) species dominate and exists as HCrO_4_^-^, Cr_2_O_7_^2-^ and CrO_4_^2-^; these have higher bioavailability, solubility and propensity for transport (Ball and Izbicki, 2004; Larsen et al., 2016), in an acidic environment, Cr(VI) does have significant Eh (1.38 V), indicating its significant oxidizing propensity (Shadreck, 2013). By influencing its chemical speciation, soil pH significantly influences Cr geochemical activity (Amin and Kassem, 2012). Soil pH determines the chemical form of Cr in soil solution and controls the balance between solubility, adsorption and desorption of Cr in soil (Ertani et al., 2017). A decrease in soil pH causes the mobilization and release of Cr(III), while an increase in soil pH leads to formation of Cr(VI) in soil (Dias-Ferreira et al., 2015). Only at pH 5.5, Cr(III) have quite a poor stability (Kabata-Pendias, 2010). Cr(III) almost fully precipitates above the pH, and thus its compounds are known to be extremely stable in soil. In contrast, Cr(VI) is highly volatile in soil, and is present in acidic and alkaline pH environments (Kabata-Pendias, 2010). Apart from directly affecting the Cr speciation, pH also influences the chemical and mineralogical properties of soil such as CEC surface charge and Eh, thereby regulating the transport, solid phase fractionation and redox behavior of Cr (Xu et al., 2020). Soil Organic matter plays an important role in determination of Cr bioavailability in soil through oxidation/reduction and adsorption/desorption (Ao et al., 2022). It binds metals in soil and performs as a transporter of Cr and several other heavy metals, reflecting soil and deposits as metals and OM storage association (Eckbo et al., 2022). Soil OM controls the Cr bioavailability and speciation through three key mechanisms (adsorption, direct and indirect reduction) (Xia et al., 2019). (1) Soil OM has a higher CEC and can form simple organic molecules and humic substances with Cr ions in soil (Schaumann and Mouvenchery, 2018). (2) Dissolved organic carbon acts as an electron donor for the reduction of Cr(VI) to Cr(III) (Li et al., 2020). (3) Soil OM drives microbial growth and creates reducing conditions that indirectly stimulate the biological reduction of Cr(VI) in the soil (Wang et al., 2019). X-ray absorption near edge structure spectroscopy revealed that increasing soil OM favors the redox transformation of Cr(VI), resulting in prevalence of reduction product Cr(III) (Jardine et al., 2013). Microorganism multiplication in soils with high OM levels creates a lowered state and modifies the soil Eh to decrease harmful Cr(VI) organisms to less harmful. Numerous organic modifications (plant tissue, black carbon, compost, farm-yard manure, and poultry manure) are widely utilized in remedial and soil restoration procedures (Kanchinadham et al., 2015).

## 6 Cr uptake and translocation in plants

In plants, the mechanism of Cr uptake is yet to be discovered. Cr is a non-essential mineral with no specialized mechanism for absorption and is also reliant on Cr speciation (Adhikari et al., 2020). The contact between roots and soil is the first interaction for uptake of Cr by plants and the uptake by plant rootsis based on plant type and Cr speciation [Cr(III) and Cr(VI)] (Shahid et al., 2017). In addition, soil pH, Cr content, salinity, and the availability of dissolved salts also influence Cr uptake in aqueous media (Babula et al., 2008). Furthermore, studies have shown that the creation of Cr-organic ligand complexes improves Cr absorption in plants (Hao et al., 2022). In various plant species, uptake of Cr takes place *via* the same carriers as for essential ions for plant metabolism (Ding et al., 2019). In plant species, the oxidation state of the Cr ions, and the concentration of Cr in the growth media influence the distribution and translocation of Cr within plants (Shahid et al., 2017). Plants can take up both Cr(III) and Cr(VI) through epidermal root cells, but there are significant differences in the pathways and efficiency of their entry into cells. Cr(VI) is more easily taken up by plants as compared to Cr(III) due to higher water solubility and higher transmembrane efficiency (Aharchaou et al., 2017). The uptake of Cr(III) is a passive process with no use of energy, most Cr(III) is taken up by roots through the same carriers as for essential elements (Singh et al., 2013). However, the routes of Cr(III) entry into cell are not well established. The uptake of Cr(VI) is an active process and relies on phosphate or sulfate carriers owing to similarity in structure (Ding et al., 2019). Cr mobility in plant roots is low as compared to other heavy metals (Sharma et al., 2020). Thus, the concentration of Cr in the roots can be 100 times more than that of the shoots (Gupta and Sinha, 2006). Cr may be sequestered in the vacuoles of root cells as a protective strategy, resulting in increased Cr accumulation in roots (Mangabeira et al., 2011). As a result of this mechanism, plants have some inherent tolerance to Cr toxicity (Srivastava et al., 2021). Furthermore, Cr translocation from the roots to the aerial shoots is quite limited, and it is highly dependent on the chemical form of Cr within the tissue (Shahid et al., 2017). Cr(VI) is changed to Cr(III) in plant tissues, which tends to adhere to cell walls, preventing Cr from being transported further into plant tissues (Kabata-Pendias and Szteke, 2015).

Cr(III)Cr(VI)The activation of ferric reductase enzymes in roots leads to active transport of Cr(VI) and results in its rapid conversion to Cr(III) (Zayed et al., 1998). This transformed Cr(III) attaches to the cell wall, preventing it from transporting through the various plant tissues (Shanker et al., 2009). Increased MSN1 (a potential yeast transcriptional activator) production resulted in increased Cr and S absorption and tolerance in transgenic tobacco (*Nicotiana tabacum*) (Shahid et al., 2017). In the transgenic Indian mustard plant, Cr(VI) stress promotes the expression of SHST1 gene, a high affinity sulfate transporter located on the plasma membrane that mediates Cr(VI) uptake by roots (Lindblom et al., 2006). Studies on sulfate transporters confirmed that Sultr1;2 gene knockout in Arabidopsis thaliana inhibits Cr(VI) uptake rate, whereas its over expression in rice significantly increases the Cr(VI) uptake by roots (Xu et al., 2021).

## 7 Effect of chromium toxicity in plants

**Figure 2:**
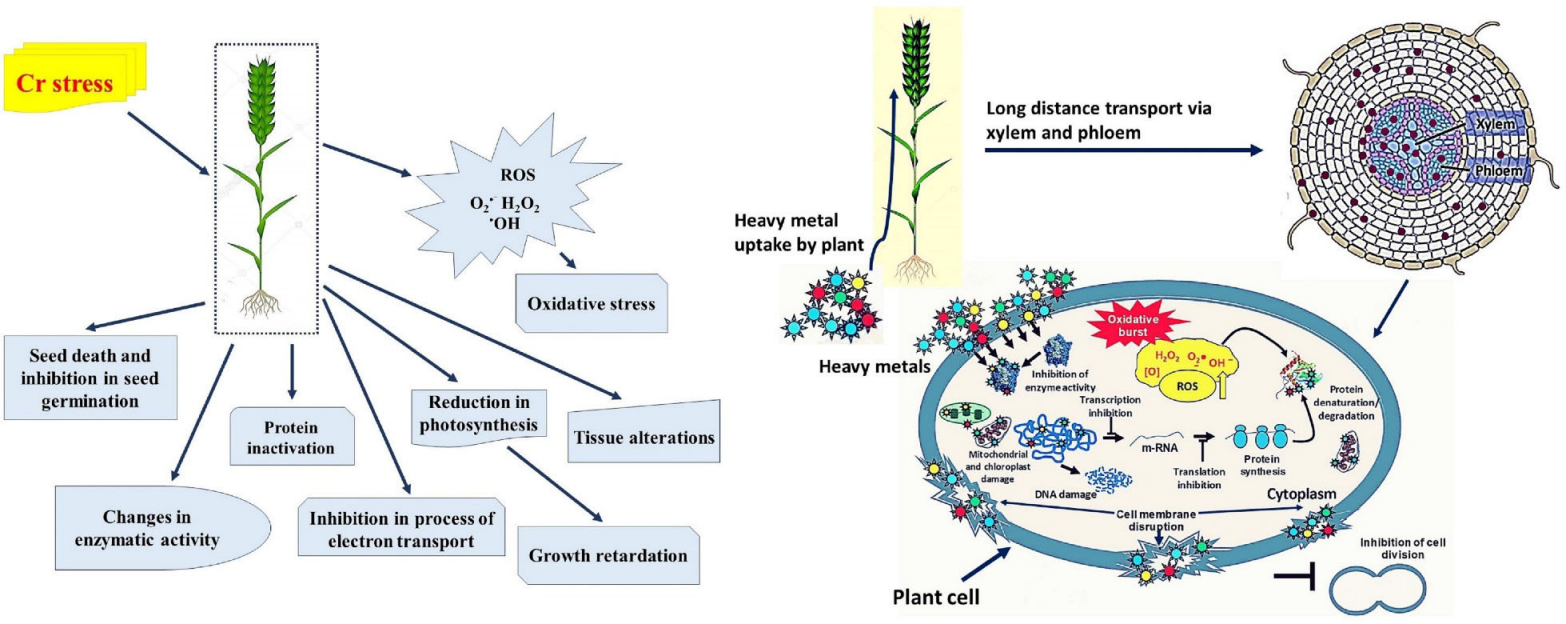
Schematic representation from the sequestration of chromium (Cr) into a plant cell to the plant’s death, through a series of events. Cr toxicity decelerates photosynthesis by preventing seedling establishment and root growth, which in turn slows down essential nutrient and water uptake. Moreover, toxicity of Cr alters photosynthetic pigments content in plant leaves, and these alterations typically result in chlorosis and necrosis of the leaves. In addition, to decreasing membrane integrity, high Cr stress also causes the loss of osmolytes and cell turgor pressure, which causes stomatal closure impacting overall osmoregulation. Additionally, Cr toxicity disrupts the equilibrium between the generation of reactive oxygen species (ROS) and the antioxidant defense system, which causes ROS to build up and cause oxidative damage to cellular organelles. DNA damage, protein and lipid synthesis, lipid peroxidation, enzyme activity, and impaired cell division are all affected by the formation of ROS, which ultimately leads to plants death (Shanker et al., 2005; Rizvi et al., 2020).

Cr may enhance plant development at low concentrations and hinder plant growth at higher concentrations, according to some research, even though there is no concrete proof to substantiate its positive participation in plant metabolism (Ao et al., 2022). In plants, higher concentration of Cr significantly affects various biochemical and morphological parameters i.e., reducing seed germination, plant biomass, photosynthetic efficiency, root damage, and eventually causes plant mortality (Zayed and Terry, 2003; Zaheer et al., 2015; **Figure 2**; **Table 1**). Excess amounts of Cr can cause stunted growth of the plant (Faisal and Hasnain, 2005a). Essential nutrients and Cr interaction can disturb the uptake pattern of various essential nutrients calcium (Ca^2+^) and magnesium (Mg^2+^) in the plant because of the interaction of Cr with soil (Zupančič et al., 2004). Moreover, agricultural soils with high levels of Cr contamination adversely affect the crop yield (Kanwal et al., 2014; Adrees et al., 2015a). Throughout the growth cycle, plants are sensitive to Cr toxicity, and detailed information about the toxic effect of Cr on morpho-physiological and biochemical parameters and toxicity mechanisms is highlighted below.

**Table 1:** Effect of chromium stress on yield of some representative field crops.

Plant species	Cr concentration	Experiment type	Yield reduction (%)	References
Cauliflower	0.5 mM	Pot experiment	50	Chatterjee and Chatterjee (2000)
Sunflower	60 mg kg^-1^	Pot experiment	52	Fozia et al. (2008)
Spinach	150 mg L^-1^	Pot experiment	45.1	Deepali (2009)
Pea	0.4 mM	Pot experiment	27.6	Tiwari et al. (2009)
Chickpea	67.5 mg kg^-1^	Pot experiment	15.3	Wani and Khan (2010)
Spring barley	150 mg kg^-1^	Pot experiment	31	Wyszkowski and Radziemska (2010)
Paddy rice	200 mg L^-1^	Field experiment	37.5	Sundaramoorthy et al. (2010)
Canola	3.49 mg kg^-1^	Pot experiment	21	Ahmad et al. (2011)
Wheat	160 mg kg^-1^	Pot experiment	41.03	Parmar and Patel (2016)
Oat	12.95 mg kg^-1^	Pot experiment	44	Wyszkowski and Radziemska (2013)
Okra	30.46 mg kg^-1^	Pot experiment	50	Maqbool et al. (2015)
Maize	0.15 mM	Pot experiment	26	Anjum et al. (2017)
Mustard	100 mg L-1	Pot experiment	27	Kumar et al. (2020b)
Wheat	50 mg kg^-1^	Pot experiment	27	Seleiman et al. (2020)
Okra	2.53 mg kg^-1^	Pot experiment	46.91	Zeeshan et al. (2021)
Tomato	1.5 mM	*In vitro* culture	50	Hafiz and Ma (2021)
Wheat	200 mg kg^-1^	Pot experiment	58.6	Ahmad et al. (2022)

### 7.1 Germination and stand establishment

Considering that seed germination is the first physiological activity that Cr affects, a seed’s capacity to germinate in a medium containing Cr would be an indication of how tolerable it is to this metal (Shanker et al., 2005; Rath and Das, 2021). Symptoms of Cr phytotoxicity comprise the early development of seedling or impediment of seed germination, suppressed root growth, and leaf chlorosis. Cr prominently reduced the seed germination of different plants such as vegetables cauliflower (*Brassica oleracea* L.), citrullus (*Citrullus vulgaris*), and crops, wheat (*Triticum aestivum* L.), barley (*Hordeum vulgare* L.), and maize (*Zea mays* L.) (Shahid et al., 2017; Ao et al., 2022). It was noted that higher toxicity of Cr in soil reduced the germination rate of jungle rice (*Echinochloa colona*), bush bean (*Phaseolus vulgaris*), alfalfa (*Medicago sativa*), and sugarcane (*Saccharum officinarum*) by 25%, 48%, 23%, and 57%, respectively as compared with control (Shanker et al., 2005).

According to several investigations, with an increase in Cr concentration in the external medium i.e., soil/nutrients solution, the DNA content of bean seedlings gradually improved and as a result, the DNA content followed a trajectory that was the opposite of the radical expansion (DalCorso, 2012). Higher concentrations of Cr significantly minimized the bean roots by interfering the cell division process in roots (Zeid, 2001; Singh et al., 2013). During seed germination, accumulated reserve materials like proteins and starch are hydrolyzed to produce precursors like sugars and amino acids for the development of embryo axis as well as substrates for different metabolic processes (Ao et al., 2022). Additionally, when the Cr content gradually increased, the activity of the α- and β-amylases of the developing seeds decreased, which may be responsible for the inhibition of seed germination (Oliveira, 2012). Seed germination of black gram (*Vigna mungo*) was reduced to 50.70% with the presence of Cr(VI) contents (300 µM) in nutrient solution (Rath and Das, 2021). Singh and Sharma (2017), observed that chickpea (*Cicer arietinum*) and green bean (*Phaseolus aureus*) seed germination was decreased by 42.60 and 53.53%, respectively, when Cr was present at higher concentrations (100 mg/L). More than 90% of the 45 tomatoes (S*olanum lycopersicum*) genotypes displayed reduced and delayed germination within 14 days under 78 mg/L Cr(VI) stress, according to a recent study by Hafiz and Ma (2021).

Higher ROS production from Cr treatment may have facilitated the breakdown of stored nutrients in seeds cotyledon, which ultimately leads to changing the characteristics of cell membranes, hence results in reduced seedling germination (Shafiq et al., 2008; Shah et al., 2010). The significant reduction in seedling length under Cr stress might be due to the reduced water potential and secondary stress-causing obstructed nutrient absorption (John et al., 2009). Because there are fewer meristematic cells in root tips than in cotyledons and shoot apex, Cr treatment also results in diminished seedling growth, particularly of roots (Rath and Das, 2021). The hydrolytic enzymes’ activity is impacted by Cr stress, depriving the radical and plumule of seed and ultimately slowing seedling growth (Stambulska et al., 2018). According to Sundaramoorthy et al. (2009), hexavalent Cr concentration even results in chromosomal abnormalities in the roots of seedling, which stimulate c-mitosis and result in extremely reduced root growth. The amylase activity of seeds under Cr stress may be inhibited, which would lead to a reduction in the transfer of carbohydrates to the germ (Stambulska et al., 2018). Additionally, Cr treatment stimulates protease activity, which results in a lower rate of seed germination or possibly seed death (Khan et al., 2020; Ao et al., 2022).

### 7.2 Uptake and interaction with other mineral elements

By altering the soil’s nutritional composition and controlling plant nutrient absorption, distribution, and transport, Cr have a significant impact on the metabolism of minerals and causes phytotoxicity in soil-plant systems (Chen et al., 2018). Cr can alter the mineral nutrition of plants in a complex way because of its structural resemblance to some critical elements (Ding et al., 2019). Researchers have focused most of their emphasis on how Cr affects the absorption and accumulation of other inorganic nutrients. Different processes are used by plants to absorb Cr (Ao et al., 2022). Both forms, i.e., Cr(III) and Cr(VI), have the potential to obstruct the uptake of several other ionically related ions, including Fe and S. Both Cr(III) and Cr(VI) have been reported to interfere with macronutrient elements (Ca, K, Mg, N, P, and S) and trace elements (Cu, Fe, Mn, Si, and Zn) through competitive uptake, even though the methods and pathways by which plants absorb Cr(III) and Cr(VI) differ (Ding et al., 2019; Askari et al., 2021; Ashraf et al., 2022a). Complex barriers caused by Cr prevent plants from absorbing essential minerals. According to Shahid et al. (2017), the existence of Cr and critical nutrients in soil and plant cells may be the cause of their antagonistic interactions and competitive absorption. Recent studies reported that excessive Cr toxicity minimizes adsorption sites and forms insoluble/low-bioavailable compounds in rhizosphere soil, which prevents the accumulation of vital nutrients including Ca, Cu, Fe, Mg, P, S, and Zn (De-Oliveira et al., 2016; Chen et al., 2018). The absorption of essential nutrients (such as N, P, K) in paddy irrigation reduced with an elevation in the level of Cr(VI) (Sundaramoorthy et al., 2010). In addition to Cr toxicity, a reduction in Fe content in leaf tissue indicates Cr(VI) involvement in Fe supply, leading to instability in Fe metabolism instability (Gopal et al., 2009).

This reduced uptake of nutrients might be occurred because of the decrease in root development and restriction of root penetration under Cr stress, or because of the reduction in translocation of essential elements (Shahzad et al., 2018; Sharma et al., 2020). Therefore, Cr(VI) competitive binding to common carriers may decrease the absorption of several nutrients. The suppression of plasma membrane H+ ATPase could be a possible explanation for the lower absorption of many of these elements in Cr stressed plants (Shanker et al., 2005). Additionally, the significant Cr buildup in the plant cell wall may harm the plasmodesmata, which serve as crucial channels for the transport of mineral nutrients, resulting in an imbalance in their metabolism (Kitagawa et al., 2015).

### 7.3 Plant water relations

The detrimental consequences of Cr concentrations cannot be precisely predicted in soil and surface water (Waseem et al., 2014). Plant roots serve the primary purposes of absorbing inorganic and organic nutrients, and water, protecting and anchoring the plant body to the ground, storing nutrients, and promoting vegetative reproduction (Rucińska-Sobkowiak, 2016). These organs typically contain higher Cr concentrations than in the above-ground plant and are known to be the first points of contact with harmful metals like Cr ions (Shanker et al., 2005; Burkhead et al., 2009). Accumulation of Cr ions in tissues may influence soil water absorption and tends to lower the water content in plant roots (Kumar et al., 2016). The direct involvement of Cr ions with the guard cells or the early effects of Cr buildup on plant parts (such as stems and roots) are what induce stomata to close (Ahmed et al., 2016). It is believed that Cr’s effects on water supply in soils, root development, reduced water absorption, and other harmful effects are distinct from its influence on the connection between plants and soil water (Chow et al., 2018). The osmotic ability of soil solution in Cr-enriched soils may be less than that of root cell sap (Vernay et al., 2007). In these circumstances, osmotic pressure, and soil solution will significantly restrict plant water absorption levels (Vernay et al., 2007; Rucińska-Sobkowiak, 2016). When the toxic metal i.e., Cr concentration hits the 10^-3^ M threshold level, osmotic pressure is thought to exist (Levitt, 1972). Adjustments to endogenous factors, such as root structure and morphology, are more likely to influence plant water absorption indirectly (Kumar et al., 2016). After being exposed to Cr, green amaranth (*Amaranthus viridis*) showed a substantial decrease in total root area (Sampanpanish et al., 2006). Reduced root hair surface, primary root elongation, increased root dieback, and poor secondary development is abnormalities in Cr-stressed plants that affected how water and plants interacted in the soil (Shanker et al., 2005; Chow et al., 2018). In epidermal and cortical cells of bush bean plants unveiled to Cr, there was impaired turgor and plasmolysis (Vazques et al., 1987). According to Gopal et al. (2009), Cr(VI) inhibits the physiological water supply, as evidenced by a drop in leaf water capability and elevation in diffusional stiffness in spinach leaves, implying that they are growing under water stress. Cr-induced structural changes reduce plant ability to acquire water in the soil and cause insufficient root-soil interaction (Ertani et al., 2017). A broad range of water-related changes is brought about by Cr exposure throughout the entire plant. Reduced water absorption and restriction of short-distance water transport in the apoplast and symplast pathways are effects of Cr toxicity in roots (Srivastava et al., 2021). Additionally, the apoplast’s resistance to water flow is increased by the thickening of the cell wall brought on by Cr ions or other incrusting substances within cell walls (Bhalerao and Sharma, 2015). The inhibition of aquaporin functions and variations in protein expression is most likely to blame for the impaired water transport through the membrane (Ullah et al., 2019a). Such changes affect the flow of water *via* the vascular system and reduce root sap exudation (Chen et al., 2010). Long-distance water transfer is reluctant, which causes a reduction in leaf water and, as a result, a water deficit in leaves (Shahid et al., 2017). Events that enhance plants’ capacity to retain water include a quick fall in root vacuolization, osmotic ability, and alternation in the tissues of stems and leaves (Srivastava et al., 2021; Ao et al., 2022).

### 7.4 Plant root and shoot growth

Cr has a significant impact on root growth and development in addition to seed germination (Shahid et al., 2017). The roots, which are a major organ for nutrient uptake and are consequently linked to Cr uptake, act as a major source of Cr toxicity in plants (Srivastava et al., 2021). A considerable reduction in root length of sour orange (*Citrus aurantium*) seedlings was discovered while conducting an experiment in a greenhouse experiment, under doses of 200 mg/kg Cr(III), (Shiyab, 2019). In water lettuce (*Pistia stratiotes*), Cr promotes root length, width, and laminal length at low concentrations (0.25 mg L^-1^) when compared to controls, but at higher concentrations (2.5 mg L^-1^), the root length was observed to be reduced (Kakkalameli et al., 2018). Similarly, it was observed that Cr toxicity minimized the shoot length of oats (*Avena sativa*) by 41% as compared to control (Shanker et al., 2005). The growth of lateral roots and the quantity of secondary roots are further effects of Cr (Mallick et al., 2010; Srivastava et al., 2021). Root cell division may have decreased because of the Cr-induced reduction in root length. Cr(VI) prevents plants from absorbing nutrients and water, which shortens roots and reduces cell division (Shahid et al., 2017). Treatment with Cr(VI) in maize (*Zea mays*) resulted in shorter and fewer root hairs, as well as a brownish color (Mallick et al., 2010). Even various studies claimed that the cell cycle extended when exposed to Cr toxicity (Sundaramoorthy et al., 2010). According to Zou et al. (2006), green amaranth (*Amaranthus viridis*) root tip cells had their mitotic index reduced because of exposure to Cr.

Another growth metric that is frequently impacted by Cr exposure is plant stem growth (Ding et al., 2019). The shoot length of sunflower (*Helianthus annus*) was observed to decrease when Cr(VI) content increased (Fozia et al., 2008). Similarly, when the soil’s Cr(III) concentration was raised in sour orange (*Citrus aurantium*) the shoot length decreased by 90.4% at 200 mg kg^-1^ of Cr (Shiyab, 2019). After being exposed to 600 mg kg^-1^ Cr(III), tea (*Camellia sinensis*) developed a short stem that grew slowly (Tang et al., 2012). According to Lukina et al. (2016), Cr(VI) toxicity (1000 mg kg^-1^) in 32 species had a negative impact on 94% of the species’ stem growth. The Cr-reduced root growth and development, which results in decreased water and nutrient transfer to the above-ground plant components, may be the cause of the decreased stem growth and height (Srivastava et al., 2021). Additionally, increased Cr transport to shoot tissues may directly interact with delicate plant tissues (leaves) and functions (photosynthesis), affecting shoot cellular metabolism and resulting in a shorter plant (Sharma et al., 2020).

### 7.5 Oxidative damages

In general, trace metal stress plants by oxidizing them either directly or indirectly by producing reactive oxygen species (ROS) (Qianqian et al., 2022). Cr toxicity causes oxidative damage in plants through overproduction of ROS such as O^2-^, H_2_O_2_, and OH^-^ (Shahid et al., 2017; Basit et al., 2022). The process of reducing Cr(VI) to lower oxidation states is the root cause of Cr toxicity, where only ROS are produced (Shanker et al., 2005; Shahzad et al., 2018). Wakeel et al. (2020) reported that when Cr(VI) is radical reduced, the unstable intermediates i.e., Cr(IV) and Cr(V), which contribute to the generation of ROS, are created. Various plant organelles, such as mitochondria, peroxisomes, and chloroplasts, create these ROS as by-products of diverse metabolic activities (Srivastava et al., 2021). The primary causes of ROS generation in plant organelles i.e., mitochondria and chloroplasts are the inhibition of CO_2_ fixation and excessive decrease of the electron transport chain (Ao et al., 2022). Furthermore, the production of ROS is caused by the leakage of electrons from O_2_ caused by electron transport activity in mitochondria, peroxisomes, and chloroplasts (Anjum et al., 2016b). Cr toxicity in plants tends to share electrons, sulfhydryl groups in proteins establish covalent interactions with redox-inactive minerals (Anjum et al., 2017). Numerous studies have been reported showing a dramatic escalation in ROS (Sharma et al., 2019) with an increase in malondialdehyde (MDA) content with Cr toxicity (Adrees et al., 2015b). A pivotal part as signaling pathways molecules and mediators of responses to cellular metabolic disturbance, environmental stimuli, pathogen infection, various developmental stimuli, and a variety of biological and physiological responses are played by plants under normal circumstances when appropriate concentrations of ROS are present (Waszczak et al., 2018). However, the overproduction of ROS in plants results in disruption of cell homeostasis, cell membrane or protein fragmentation, DNA strand breaks, deactivation and degradation of genetic material, and harm to photosynthetic pigments (Srivastava et al., 2021; Ao et al., 2022). Similar findings were observed by Ullah et al. (2019b), who reported that increased ROS generation in plants with Cr toxicity results in oxidative damage, inflicting damage to DNA, lipids, pigments, and proteins, and stimulating the lipid peroxidation functions. These effects inhibit plant growth by preventing cell division or inducing cell death, which lowers biomass production (Wakeel et al., 2020). According to Shahid et al. (2014), the duration of exposure, Cr content, plant species, stage of development, level of stress, and particular organs all affect how hazardous Cr-induced ROS are for plants

### 7.6 Antioxidant defense system

Complex defense approaches, including non-enzymatic and enzymatic antioxidants, have evolved to prevent oxidative damage to plant cells (Semchuk et al., 2009; Ashraf et al., 2021; Zulfiqar et al., 2021). As with many other metals, excess Cr can promote the development of ROS and generally increases the activity of anti-oxidative enzymes (**Table 2**). Activities of enzymatic antioxidants such as catalase (CAT), glutathione peroxidase (GPX), ascorbate peroxidase (APX), peroxidase (POD), glutathione reductase (GR), superoxide dismutase (SOD), dehydroascorbate reductase (DHAR), glutathione-S-transferases (GST), monodehydroascorbate reductase (MDHAR), and non-enzymatic antioxidants such as glutathione (reduced form, GSH, and oxidized type, GSSG), ascorbic acid (AsA), and phenolic metabolites were significantly increased under Cr toxicity to minimized the counter effects of ROS production in plant metabolic processes (Shahid et al., 2017; Jan et al., 2020). Cr exposure was found to increase the content of GSH and AsA, while the concentration of phenolic contents was depleted (Panda, 2007). Moreover, non-enzymatic antioxidants that control the levels of ROS in cells, such as tocopherols, carotenoids, GSH, proline, and AsA are regarded as moderators of oxidative damage (Adrees et al., 2015a). Antioxidant capabilities can also be found in other low-molecular-weight substances such as tocopherols, carotenoids, and phenols (Akyol et al., 2020; Ao et al., 2022). However, their antioxidants’ activity and availability are dependent on secondary metabolites’ capacity to synthesize specific compounds, which varies widely among different plant species (Akyol et al., 2020).

**Table 2:** Effects of chromium stress on activities of different antioxidant enzymes and lipid peroxidation in different plants.

Plant species	Enzymes (%)	Culture	LPO indicator (%)	Cr exposure level	Exposure duration (days)	References
Rice	APX (245**↑**), CAT (35.1**↓**), SOD (31.6**↑**), POD (59.9**↓**)	Hydroponic	MDA (65**↑**)	20 µM	10	Cao et al. (2013)
Canola	CAT (39.42**↓**), SOD (42.85**↑**), POD (82.14**↑**), APX(37.5**↓**)	Hydroponic	MDA (66.67**↑**)	50 µM	2	Yıldız et al. (2013)
Radish	CAT, SOD, POD	Hydroponic	MDA	2- 8 mM	3	Sayantan (2013)
Pakchoi	CAT (37.84**↓**), SOD (47.04**↓**), POD (41.43**↓**)	Soil	MDA (48.5**↑**)	0, 50, 100 and 200 mg kg^-1^	2	Zhang et al. (2013)
Cotton	CAT (16.66**↑**), SOD (74.07**↓**), POD (48.5**↑**), APX (44.44**↑**)	Hydroponic	MDA (65.9**↑**)	0, 10, 50 and 100 µM	7	Daud et al. (2014)
Tossa jute	CAT (65.28**↑**), SOD (56.83**↑**), POD (59.13**↑**), GR (57.94**↑**)	Hydroponic	MDA (47.89**↑**)	100, 200 and 400 mg kg^-1^	7	Islam et al. (2014)
Black nightshade	SOD (13.51**↑**), POD (22.22**↑**)	Hydroponic	MDA (22.22**↑**)	0, 0.5 and 1 mM	2	UdDin et al. (2015)
Santa-maria	SOD (23.26**↑**), POD (42.85**↑**)	Hydroponic	MDA (38.46**↑**)	0, 0.5 and 1 mM	2	UdDin et al. (2015)
Rapeseed	CAT (54.54**↑**), SOD (49.37**↑**), POD (23.08**↑**), APX (57.5**↑**)	Soil	MDA (70.2**↑**)	0, 100 and 500 µM	15	Afshan et al. (2015)
Indian mustard	SOD (66.14**↑**), CAT (42.08) **↑**), POD (59.11**↑**), APX (33.15**↑**), GR (46.97**↑**), DHAR (70.38**↑**), MDHAR (71.52**↓**)	Hydroponic	MDA (50.36**↑**)	0.1, 0.3 and 0.5 mM	30	Kanwar et al. (2015)
Egg plant	APX (12**↑**), GST (38**↑**), GR (20**↑**)	Hydroponic	MDA (13**↑**)	25 µM	7	Singh et al. (2017)
Amaranth	CAT (44**↑**), SOD (50**↑**), POD (74**↑**), GST (101**↑**)	Hydroponic	MDA (108**↑**)	0, 10 and 50 µM	7	Bashri et al. (2016)
Maize	CAT (48.52**↑**), SOD (17.14**↑**), POD (36.67**↑**)	Hydroponic	MDA (126**↑**)	100 µM		Anjum et al. (2016b)
Kenaf	CAT (151.43**↑**), SOD (135.79**↑**), POD (58.46**↑**)	Hydroponic	MDA (53.51**↑**)	1.5 mM	6	Ding et al. (2016)
Rice	CAT (74.42**↓**), SOD (9.33**↓**), POD (64.91**↓**), GR (54.84**↓**)	Hydroponic	H_2_O_2_ (86.89**↑**)	100 µM	7	Huda et al. (2016)
Green gram	CAT (31.03**↑**), SOD (46.25**↑**), POD (34.21**↑**)	Hydroponic	MDA (51.67**↑**)	0, 250 and 500 µM	7	Jabeen et al. (2016)
Sunflower	CAT (70.83**↑**), SOD (75.61**↑**), POD (20.12**↑**), APX (62.5**↑**)	Hydroponic	MDA (71.43**↑**)	0, 5, 10 and 200 mM	15	Farid et al. (2017)
Wheat	CAT (40.1**↓**), APX (13.46**↑**)	Soil	MDA (16.67**↑**)	10 and 22 mg kg^-1^	30	González et al. (2017)
Barley	CAT (41.82**↓**), APX (22.5**↑**)	Soil	MDA (27.27**↑**)	10 and 22 mg kg^-1^	30	González et al. (2017)
Cauliflower	CAT (34.78**↑**), SOD (37.5**↑**), POD (35.1**↑**)	Hydroponic	MDA (63.33**↑**)	0, 10, 100 and 200 µM	7	Ahmad et al. (2017)
Sorghum	CAT (66.67**↑**), SOD (90.1**↑**), APX (80.2**↑**), GR (64.5**↑**), GST (36.5**↓**)	Hydroponic	MDA (61.67**↑**)	2, 4, 8, 16, 32 and 64 mg kg^-1^	7	Yilmaz et al. (2017)
Indian mustard	CAT (39**↓**), SOD (16**↑**), APX (28**↑**), GR (16**↑**), GPX (14**↓**), DHAR (50**↓**), MDHAR (31**↓**)	Hydroponic	MDA (101**↑**)	0.15 and 0.3 mM	5	Al-Mahmud et al. (2017)
Maize	GR (29.33**↓**)	Hydroponic	MDA (65.71**↑**)	50, 100 and 200 mg L^-1^		Adhikari et al. (2020)
Tomato	–	Petri dish	MDA (63.23**↑**)	50 µM		Khan et al. (2020)

Ascorbate peroxidase (APX), catalase (CAT), dehydroascorbate reductase (DHAR), glutathione peroxidase (GPX), glutathione reductase (GR), glutathione S-transferase (GST), monodehydroascorbate reductase (MDHAR), peroxidase (POD), and superoxide dismutase (SOD), malondialdehyde (MDA).

Plant roots with high levels of Cr(III) content, SOD increased primarily, while the quality of H_2_O_2_ displayed a discontinuous pattern for the various Cr(III) absorption, which was assumed because of heterogeneity in the activity of various peroxidases (Kováčik et al., 2013). Plant resistance may have surpassed the innate immune level for high doses of Cr in this case, resulting in the observed declines in enzyme activity. With increasing Cr(III) content, there was an increase in proline content. Usman et al. (2020), reported that giant milkweed (*Calotropis procera*) treated with Cr(VI) (20 mg L^-1^) showed enhanced activity of CAT, GR, and SOD with SOD activity being the greatest (up to 12.2 U mg^-1^). The formation of reducing agents (GSH and AsA metabolites) that catalyze the dismutation of H_2_O_2_ to O_2_^-^ and H_2_O is aided by the synergistic effects of GR, CAT, and APX and which all play critical roles in scavenging ROS (Bashir et al., 2020). When Cr metal binds to proteins, whether in the catalytic domain or elsewhere, it inhibits enzyme reactants by attaching unique functional groups to proteins, resulting in enzymatic function modifications (Gupta et al., 2010). In addition, from the enzyme, dislocation of essential cations the equilibrium of ROS in cells is disrupted by binding sites, and consequently, ROS is produced in dramatic amounts (Shahzad et al., 2016). The oxidation number of glutathione (GSH) and its constituents appear to bind and utilize Cr metal, which is important for reducing ROS (Lee et al., 2003). In addition, NADPH oxidase contributes to oxidative damage as it is associated with Cr (Pourrut et al., 2013). NADPH oxidases can consume cytosolic NADPH in the existence of Cr metal and generate free radical O_2_; it is quickly converted to H_2_O_2_ through SOD enzyme (Shahid et al., 2017). In the presence of NADPH oxidase, Cr-generated free radicals are external to the plasma membrane, where the pH is generally lower than on the interior side of the membrane (Sagi and Fluhr, 2006). The transporter membrane promotes Cr ingestion and affects the plasma membrane’s ability to produce ROS (Maiti et al., 2012). However, the underlying molecular mechanisms of scavenging ROS by antioxidants and non-enzymatic antioxidants are yet unknown and need more research.

### 7.7 Photosynthetic activity and yield formation

Phytotoxicity of Cr adversely affects various metabolic processes i.e., CO_2_ fixations, electron transfer, photophosphorylation, and enzyme concentration, which directly impairs photosynthesis (Anjum et al., 2017; Sharma et al., 2020; Ashraf et al., 2022b). Taken to be critical indices that measure plant photosynthesis under Cr stress are photosynthetic rate, photosynthetic pigments, and photochemical efficiency (Ma et al., 2016). Cr is a potent inhibitor of plant photosynthesis, according to numerous studies (Shanker et al., 2005; Shahzad et al., 2016; Bashir et al., 2020). According to Mathur et al. (2016), Cr toxicity prevents CO_2_ fixation, electron transfer, enzyme activity, and photophosphorylation in plants. This destroys the photosynthetic apparatus, specifically light-harvesting complex II, PSI, and PSII, and prevents the production of Calvin cycle enzymes (responsible for ATP production) (Sinha et al., 2018). In a study, Anjum et al. (2016a) found that maize plants exposed to Cr stress had significantly lower the levels of net photosynthesis, chlorophyll contents, gas exchange capacity, transpiration rate, water use efficiency, and stomatal conductance. The degradation of photosynthetic pigments caused by exposure to the high concentration of Cr leads to reduction in light-harvesting capacity (Handa et al., 2018b; Srivastava et al., 2021). Net photosynthetic rate (Pn) and chlorophyll content in wheat (*Triticum aestivum*) were decreased as Cr exposure period gradually increased (Srivastava et al., 2021). Cr prevents mitochondrial electron transport in higher plants, which increases the production of ROS and causes chloroplast modifications, pigment changes, and oxidative stress (Sharma et al., 2016). One of the crucial plant parts involved in photosynthesis is the leaf and total leaf area (Srivastava et al., 2021). In rice (*Oryza sativa*) the Cr(VI) toxicity reduced the number of leaves per plant by 50% while significantly affecting the overall leaf area and photosynthesis activity of plant (Sundaramoorthy et al., 2010). Under 3.4 mM Cr(VI) toxicity in nutritional media, smooth mesquite (*Prosopis laevigatar*) was shown to have fewer leaves that significantly affect the chlorophyll content and photosynthesis activity of plant (Buendía-González et al., 2010). Furthermore, it was shown that Cr toxicity significantly decreased the leaf’s net photosynthetic rate, transpiration rate, stomatal conductance, and intercellular CO_2_ concentration, of sunflower with reductions of 36%, 71%, 57%, and 25%, respectively (Sharma et al., 2020).The first requirement for large plant yields is high plant biomass (Shahid et al., 2017). Cr is known to have negative impacts on several physiological and metabolic processes, which compromises plant production and yield equally (Ali et al., 2015). Various studies highlighted that Cr phototoxicity results to minimize plant biomass and yield of melon (*Cucumis melo*) (Akinci and Akinci, 2010), wheat (*Triticum aestivum*) (Adrees et al., 2015a), french bean (*Phaseolus vulgaris*) (Sharma et al., 2016), okra (*Hibiscus esculentus*) (Amin et al., 2013), turnip mustard (*Brassica campestris*) (Qing et al., 2015), Arabidopsis (*Arabidopsis thaliana*) (Ding et al., 2019), common duckweed (*Lemna mino*r) (Reale et al., 2016), wheat (*Triticum aestivum*) (Ali et al., 2015), barley (*Hordeum vulgare*) (Ali S. et al., 2013) maize (*Zea mays*) (Anjum et al., 2017), cotton (*Gossypium hirsutum*) (Farooq et al., 2016), makoi (*Solanum nigrum*) (UdDin et al., 2015). In plants, higher concentration of Cr significantly affects various biochemical and morphological parameters i.e., minimized nutrient and water uptake, reduction in cell division, nutrients imbalance (translocation and uptake), the inefficiency of inorganic nutrient uptake by plant, higher oxidative stress, and ROS formation, oxidative stress damage to sensitive cell organelles such as chlorophyll, mitochondria, lipids, proteins, and reduction in photosynthesis activity that results to minimize the growth, biomass, yield of plant (Shanker et al., 2005; Shahid et al., 2017; Ao et al., 2022). At the cellular, molecular, organ, and plant levels, each of these elements, alone or in combination, have an impact on plant growth, development, and yield (Shahid et al., 2017). However, the type of plant and chemical speciation of Cr will determine which of these factors will be more severely impacted. The impact of Cr on plant development, however, differs depending on the variety of plants. In general, transgenic and hyperaccumulator plants have a lot of potential for Cr tolerance and selective accumulation (Sarangi et al., 2009).

### 7.8 Enzymatic activity

Cr stress can stimulate potentially three forms of metabolic changes in plants: (i) modification in the synthesis of organic pigments facilitates the growth and development of plants (e.g., anthocyanin, and chlorophyll (Shanker et al., 2005; Shahid et al., 2017); (ii) enhanced the synthesis of metabolites (e.g., ascorbic acid, and glutathione) as a direct reaction to Cr stress that will affect the plants (Srivastava et al., 2021); and (iii) modifications in the metabolic-pool to channelize the synthesis of new biochemically associated metabolites that will confer tolerance or resistance to Cr stress (e.g., histidine and phytochelatins) (Shanker et al., 2005; Ao et al., 2022). Initially at germination stage, toxicity of Cr significantly reduced the activity of gibberellin (GA) and enhanced the activity of abscisic acid (ABA) (major factor of seed dormancy), which lead to seed imbibition and reduced germination rate (Seneviratne et al., 2019). Similarly, according to Yan et al. (2014) hydrolyzing enzymes secreted by the aleurone layer of seeds are crucial for seed germination. By releasing food reserves from the endosperm, enzymes i.e., acid phosphatases (ACPs), α-amylases, and proteases promote effective seedling establishment and growth (see section 5.1). Acid phosphatase, α-amylase, and alkaline phosphatase activity were decreased in the endosperm of cereals i.e., wheat, oat, barley, and maize seeds when Cr was present (Seneviratne et al., 2019). In addition, the enzymes involved in the assimilation of important nutrient nitrogen i.e., nitrogenase, nitrate reductase, nitrite reductase, glutamine synthetase, glutamate synthase, glutamate dehydrogenase were significantly reduced with the contamination of Cr in plants (Sangwan et al., 2014). Deficiency of nutrients in plants due to Cr toxicity results into degradation of various amines, alkaloids, pigments, vitamins, coenzymes, nucleic acids, and nucleotides as nutrients are structural component of these organelles (Shanker et al., 2005; Sangwan et al., 2014). Similarly, the activities of enzymes involved in photosynthesis NADP-malic enzyme (NADP-ME), pyruvate, phosphate dikinase (PPDK), and Phosphoenolpyruvate carboxylase (PEPC), plant respiration i.e., α-ketoglutarate dehydrogenase and isocitrate dehydrogenase, and gene transcription i.e., RNA polymerase are significantly reduced in various plants due to phototoxicity of Cr.

## 8 Remediation of Cr contaminated soils

The concentration of metals in polluted soils is affected by multiple chemical and biological attributes (Alengebawy et al., 2021). Soils preserve heavy metals by adsorbent, crystallization, and chelation; nevertheless, such interactions restrict their mobility and bioavailability (Yan et al., 2020). However, the implementation of chemical processes, such as organic and inorganic modifications in field can complement this natural attenuation process (Mench et al., 2006; Shahid et al., 2017). These technologies generally minimized the availability of Cr, boost the fertility of the soil, and increase plant growth (Gavrilescu, 2022). Organic amendments (compost) possess a significant proportion of humified organic material and may restrict the availability of Cr in the soil, even though they allow vegetation to be regenerated (Lwin et al., 2018). On the other hand, phosphate fertilizers are useful in metal inactivation through the creation of stable mineral phosphate within the inorganic amendments (Ahmad et al., 2019). Biological options, particularly phytoremediation, have been considered reliable, ecologically acceptable, and cost-effective replacement to physicochemical approaches for the restoration of depleted environments. Various physicochemical activities that can be used to eliminate Cr-polluted environments include ionization, precipitation, reverse osmosis, evaporation, and chemical reduction (Roy and Bharadvaja, 2021). Moreover, there are numerous issues linked with these processes, like permeate flux, inflated prices, high energy consumption, and low extraction efficiency shows that these are less significant in industry. In general, the main considerations in choosing an acceptable treatment to eliminate metals are technological applicability, eco-friendly, and cost-effectiveness (Acheampong et al., 2010).

### 8.1 Phytoremediation

Phytoremediation is a process in which plants are used for remediation of polluted soils and considered an eco-friendly and green approach (Ali H. et al., 2013; Srivastava et al., 2021). There are various strategies associated with phytoremediation techniques including phytoextraction, rhizofiltration, phytovolatilization, biotransformation, rhizdegradation, phytostabilization, and phytorestoration (Yan et al., 2020). Phytoextraction is focused on the ‘hyperaccumulation’ process, and phytostabilization is focused on the surface complexation mechanism and both are involved in metal affinity phenomena (Xu et al., 2012). Phytoextraction and phytostabilization are two of those practically and economically viable solutions for treating metal-polluted soils (Kuiper et al., 2004). Biotransformation is another term for phyto-transformation. That is the separation of pollutants absorbed by plants *via* internal metabolic pathways or the segmentation of pollutants just outside of the plant because of plant-generated chemicals (such as enzymes). Plant absorption and metabolism are the primary components, which result in plant deterioration. The uptake of contaminants by plant roots and its conversion to a gaseous state, and release into the atmosphere is referred as phytovolatilization. Volatilization through leaves (ITRC, 2009) is the phytovolatilization process. Degradation by plant rhizospheric microorganisms is the method referred as rhizodegradation (Mench et al., 2009). This ecologically accepted technology is successfully used to fix soils that are polluted by various contaminants. Furthermore, phytoremediation is increasingly used as a technical alternative to treat contaminated water in various forms of wetland treatment (Zhang et al., 2010). In crux, phytoremediation is a feasible, socially, and economically suitable, and eco-friendly solution for the soils polluted with Cr. Nonetheless, to counteract the health risks due to Cr concentration in edible parts of food crops, the proportion of Cr in edible parts of food crops should be closely scrutinized.

### 8.2 Microbe-assisted remediation

**Figure 3:**
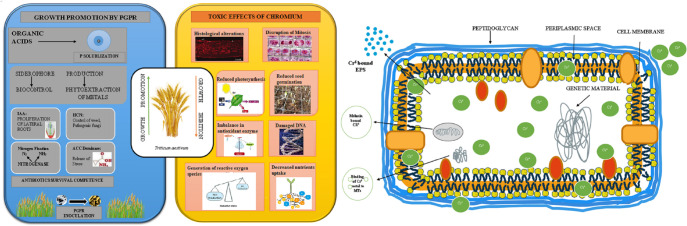
A schematic illustrating how rhizobacteria that encourage plant growth might boost growth and reduce the damaging effects of chromium (Cr) on plant. The removal/detoxification of Cr ions by active biomolecules i.e., secretion of melanin, metallothionein (MTs), and polymeric substances (EPS), released by rhizobacteria strains under Cr stress (Rizvi et al., 2020; Ao et al., 2022).

Several methods of metal remediation have been used to address the harmful impacts of metal contamination, including physical, chemical, and biological processes, to inactive specific hazardous metals from the atmosphere (Marques et al., 2011). Microbial remediation has gained significant attention among different biological remediation methods because of its cost-effectiveness, higher efficacy, and non-expendable technologies (Malaviya and Singh, 2014; Fernandez et al., 2018). Some of the microbes that tolerate Cr establish ability to minimize the toxicity of Cr(VI) concentration from the atmosphere and thus play a prominent role in the remediation of Cr(VI) (**Table 3**; **Figure 3**). Many investigations on the collection and profiling of distinct Cr-lowering microbial strains of bacteria have been published in last few years (*Pseudomonas* spp., *Bacillus* spp., *Enterobacter* spp., *Acinetobacter* spp,.), fungi (*Aspergillus* spp., *Penicillium* spp., *Rhizopus* spp.), and yeast (*Candida* spp., *Saccharomyces* spp.) (Chen et al., 2016).

**Table 3:** Biosorption of chromium by application of different microbes.

Microbial group	Microbial biosorbent	pH	Temperature (°C)	Time	Initial metal ion concentration (mg L^-1^)	Removal efficiency (%)	Reference
Fungi	*Saccharomyces* *cerevisiae*	5	25	3 h	90	99.6	Rossi et al. (2018)
	*Aspergillus sydowii*	5	28	7 d	50	24.9	Lotlikar et al. (2018)
	*Arthrinium malaysianum*	3	30	20 h	1000	67	Majumder et al. (2018)
	*Penicillium oxalicum* SL2		30	144 h	1000	100	Long et al. (2018)
	*Aspergillus niger* (CICC41115)	7	37	84 h	50	100	Gu et al. (2015)
	*Saccharomyces cerevisiae*	3.5	25	24	200	85	Mahmoud and Mohamed (2017)
	*Aspergillus* sp. FK1	5		7 d	557	65	Srivastava and Thakur (2006b)
Bacteria	*Acinetobacter* sp. B9	7	30	24 h	7.0	67	Bhattacharya and Gupta (2013)
	*Enterobacter cloacae* strain CTWI-06	7	37	92 h	500	94	Pattnaik et al. (2020)
	*Escherichia coli* VITSUKMW3	7.5	30	5 h	20	40	Samuel et al. (2012)
	*Staphylococcus aureus* strain K1	8	35	24	100	99	Tariq et al. (2019)
	*Bacillus subtilus* PAW3	6	35	20	100	100	Wani et al. (2018)
	*Cellulosimicrobium* *Funkei* strain AR6	7	35	120	250	80.43	Karthik et al. (2017)
	Acinetobacter sp. AB1	10	30	72 h	50	100	Essahale et al. (2012)
	Streptomyces sp. MC1	7.4	30	72 h	50	52	Polti et al. (2011)
	*Bacillus subtilis* MNU16	7	30	72 h	50	75	Upadhyay et al. (2017)
	*Pseudomonas* sp. JF122	6.5	30	72 h	2.0	100	Zhou and Chen (2016)
	*Acinetobacter guillouiae* SFC 500 – 1A	10	28 ± 2	72	10	~62	Ontañon et al. (2015)
	*B. mycoides* 2000AsB1	7	30	25 h	25	100	Wang et al. (2016)
	*Streptomyces werraensis* LD 22	7	41	7 d	250	51.7	Latha et al. (2015)
	*Arthrobacter* sp. Sphe3	8	30		45	100	Ziagova et al. (2014)

The use of plant growth-promoting bacteria (PGPB) in plants, is also regarded as a significant and environmentally acceptable method for the removal of heavy metals from soil (Fahad et al., 2014). These bacteria encourage plants to endure extreme stress and improve plant nutrition to stimulate plant growth (N, P, Fe) and release different metabolites related to stress, such as the production of phytohormones, solubilization of phosphates, and production of siderophores (Dodd and Perez-Alfocea, 2012). Several studies have documented the use of plant growth promotion (PGP) rhizobacteria for heavy metal bioremediation, like *Bacillus* sp., *Pseudomonas* sp., etc. (Ndeddy-Aka and Babalola, 2016). Microorganisms have been found to reduce hexavalent Cr through various means, either by using hexavalent Cr as the final acceptor of electrons or by releasing some dissolving enzymes (**Table 4**; Ahemad, 2015). In an experiment, Karthik and Arulselvi (2017) evaluate the effect of Cr(VI) on the plant growth-promoting properties of potential rhizobacterial strain isolated from the rhizosphere of a common bean (*Phaseolus vulgaris*). The strain AR8 was chosen from 36 rhizobacterial strains when compared to uninoculated Cr(VI) treated plants, the inoculation of *Cellulosimicrobium funkei* strain AR8 significantly improved the root length of test crops in both the presence and absence of Cr(VI). Strain AR8 could be used for growth stimulation as well as for the removal of Cr in Cr-contaminated soil because of these exceptional characteristics (**Figure 3**).

**Table 4:** Effects of plant growth promoting rhizobacteria (PGPR) on plants in Cr-contaminated soils.

Plant species	PGPR	Method of application	Amount of PGPR	Cr concentration	Effect	References
Indian mustard	*Pseudomonas* sp. PsA4, *Bacillus* sp. Ba 32	Seedling inoculation	10^8^ cell mL^-1^	281 mg kg^-1^	Increased plant growth (phytostabilization), decreased Cr content.	Rajkumar et al. (2006)
Chickpea	*Mesorhizobium* sp. RC3	Seedling inoculation	Approx. 10^8^ cell mL^-1^	136 mg kg^-1^	The bio-inoculant decreased the assimilation of Cr by 14, 34 and 29% in roots, shoots and grain respectively.	Wani et al. (2008)
Sunflower	*Ochrobactrum intermedium*	Seedling inoculation	300 µg mL^-1^	300 µg g^-1^	Increased growth of plant and decreased Cr(VI) uptake.	Faisal and Hasnain (2005a; b)
Green gram	*Ochrobactrum* sp., and *Bacillus cereus*	Seedling inoculation	300 µg mL^-1^ bacterial suspension	384 µg g^-1^	Cr toxicity to seedlings is lessened from Cr(VI) to Cr(III).	Faisal and Hasnain (2006)
Common bean	*Cellulosimicrobium funkei* (KM263188)		0.024 mg kg^-1^ (garden soil) and 42.65 mg kg^-1^ (leather industrial soil)	Serial dilution (up to 10^-7^)	Increased crop production, showed tolerance to Cr(VI), produced plant growth-promoting substance.	Karthik and Arulselvi (2017)
Alfalfa	*Pseudomonas* sp.	Seedling inoculation	10^8^ CFU mL^-1^ bacterial suspension	10 mg kg^-1^	Improved alfalfa growth and antioxidant system under Cr stress and enhanced Cr(VI) phytoremediation	Tirry et al. (2021)
Green gram	*Bacillus* sp. AMP2, *Halomonas* sp. AST, *Arthrobacter mysorens* AHA, *Kushneria avicenniae* AHT, *Halomonas venusta* APA	Seedling inoculation	10 to 1000 µg mL^-1^	100 µg mL^-1^	Reduced the damaging effects of Cr on the environment, primarily on soil.	Arshad and Ahmed (2017)
Maize	T2Cr and CrP450	Seedling inoculation	10^8^ CFU mL^-1^ bacterial suspension		Improved production potential of maize, reduced oxidative stress	Islam et al. (2016)
Black gram (*Vigna mungo*)	*Pseudomonas aeruginosa* ATCC P15442 (P15)	Seed inoculation	10 mL of NBRIP broth medium inoculated with 10% bacteria cell	100 and 250 µg mL^-1^	Reduced heavy metals, soil productivity enhanced due to PGPR.	Kumar et al. (2020a)
	*Bacillus subtilis* MNU 16		2 x 10^6^ bacteria/mL bacterial suspension	50-300 mg/L	Reduced toxic form of Cr(VI) to less toxic form Cr(III), improved the efficiency of rhizoremediation of contaminated soils.	Upadhyay et al. (2017)
Common bean	*Cellulosimicrobium funkei* (AR6)			1200 µg mL^-1^	Inoculation of rhizobacteria in polluted soils could be a good approach for soil rehabilitation.	Karthik et al. (2017)
Maize	*Agrobacterium fabrum* and *Leclercia adecarboxylata*	Foliar application	10 mL of inoculum was applied along 10% sugar in 100g sterilized seeds.	50 and 100 mg kg^-1^	Chlorophyll content and nutrient concentration increased and Cr toxicity decreased.	Danish et al. (2019)
Lentil (*Lens culinaris*)	*Bacillus* sp.	Seed inoculation	10^6^-10^7^ CFU mL^-1^	500 µg mL^-1^	PGPRs protected the plants from heavy metals by producing phytohormones and antioxidant enzymes.	Fatima and Ahmed (2018)
Wheat	*Bacillus* sp.	Seed inoculation	10^7^ CFU mL^-1^	95-1180 mg kg^-1^	PGPR in combination with biochar increased root and shoot length, chlorophyll content and sugar contents, it also controlled the Cr.	Mazhar et al. (2020)
Mesquite trees (*Prosopis laevigata*)	*Bacillus* sp. MH778713	Seed inoculation	1x10^6^ UFC suspended in 1 mL of sterile distilled water	435 mg kg^-1^	Bacillus sp. is thought to be a viable option for heavy metals-contaminated soil rehabilitation.	Ramírez et al. (2019)
Wheat	180 Cr(VI) tolerant bacteria	Seed inoculation	10^7^-10^8^ CFU mL^-1^	20 mg kg^-1^	Cr concentration decreased with the application of PGPR.	Khan M.Y. et al. (2013)
Wheat	CC7 and ACC-14	Seed inoculation	10^7^-10^8^ CFU mL^-1^	0-100 mg L^-1^	Phytotoxicity was reduced by using PGPR like CC7 and ACC-14.	Rai et al. (2016)
Bajra (*Pennisetum* *glaucum* L.)	*Bacillus* sp., *Pseudomonas* sp., *Azotobacter* sp., and *Rhizobium* sp.		200 µg mL^-1^	25 to 2000 µg mL^-1^	Decreased the heavy metal contaminants present in the soil.	Saif and Khan (2017)
	*Achromobacter xylosoxidans* (LK391696), and *Azotobacter vinelandii* (LK391702)		46 µg mL^-1^ and 30 µg mL^-1^	0.2 mg kg^-1^	PGPRs reduced Cr concentration and improved plant growth.	Mohan et al. (2014)
Rice	*Bacillus* sp.		10^-3^ to 10^-7^	50 to 100 µg	Plant growth stimulation and biocontrol work together to boost vegetative and crop yields.	Karuppiah and Rajaram (2011)
Maize	PGPR LCC41, LCC81	Seed inoculation	10^8^ CFU mL^-1^ bacterial suspension	320 mg kg^-1^	PGPRs improved plant growth, and soil microbial activity and reduced translocation of Cr within plant	Silva et al. (2021)

According to Caravelli et al. (2008) the *Sphaerotilus natans* CSCr-3, a filamentous bacterium obtained from activated sludge, reduced Cr concentration up to 1.5 mM in the presence of a carbon source. This removal efficiency is significant because *S. natans* was originally recognized for its biosorption capability. Under alkaline medium, another bacterium, *Ochrobactrum* sp., was able to decrease Cr(VI). This isolate substantially tolerated and reduced Cr(VI) up to 15.4 mM. The inclusion of glucose generated a significant improvement in Cr(VI)-reduction, while the availability of sulphate or nitrate had no effect (He et al., 2009). Five Cr resistant bacterial strains with auxin biosynthesis abilities were used by Arshad and Ahmed (2017). *Halomonas venusta* APA and *Arthrobacter mysorens* AHA were determined to be the most effective isolates in terms of phytostimulatory effects on green gram (*Vigna radiata*). A huge proportion of microbial variants have been recorded for remediation of Cr(VI) using biosorption and bioaccumulation methods, such as *Paecilomyces lilacinus* (Sharma and Adholeya, 2011), *Aspergillus niger* (Srivastava and Thakur, 2006a, b), *Bacillus cereus* IST105 (Naik et al., 2012), *Zobellella denitrificans* (He et al., 2016), and *Bacillus mycoides* 200AsB1 (Wang et al., 2016). In conclusion, the use of a suitable microbial inoculum might become useful in effectively altering the soil infected with Cr.

### 8.3 Chemical remediation

*In-situ* or ex-situ complex formation through chelating substances has been used for metal extraction (Di-Palma et al., 2005; Finzgar and Lestan, 2007). The efficacy of extraction depends upon the availability of readily exchangeable ions in the soil matrix capable of forming strong complexes with minimum specific chelating agents (Di-Palma, 2009). For removal of maximum amounts of metals found in polluted soils, phytoextraction may be used, with some portion of the soil metal content freely available to plants. There are various synthetic chelating components, such as EDTA (ethylene diamine tetra acetic acid), diethylene trinitrile pentaacetic acid (DTPA), nitrile triacetic acid (NTA), pyridine-2,6-dicarboxylic acid (PDA), trans-l,2-diaminocyclohexane-N,N,N0,N0-tetraacetate (CDTA), or ethylenediamine disuccinate (EDDS) used for remediation of soil polluted with organic and inorganic contaminants. To increase the accessibility of metals in soil and the transference of metals from root to shoot, several ideas have been proposed (Meers et al., 2005). Application of chelating agents substantially improved the Cr uptake in above-ground biomass of many crops (**Table 5**). Patra et al. (2018) revealed that in Cr(VI) polluted soil, application of chelators including EDTA, DTPA, citric acid, and salicylic acid, along with metal ions, enhanced the growth of lemongrass (*Cymbopogon citratus*) and enhanced Cr bioavailability. Chigbo and Batty (2013) analyzed that the application of EDTA and citric acid reduced alfalfa (*Medicago sativa*) shoot dry matter by 55%, decreasing the soil Cr removal efficiency. The removal of Cr increased to 54.28% when the polluted soil was pre-treated with 0.01M EDTA-2Na (Xu Y. et al., 2019). Mohanty and Patra (2012) revealed the total accumulation rate for Cr was improved with the application of DTPA to rice (*Oryza sativa*) and wheat (*Triticum aestivum*), While the use of EDDHA was proven to be useful in accelerating the process of Cr accumulation in green gram (*Vigna radiata*) seedlings. The role of chelating substances in reducing the harmful impact of Cr(VI) is demonstrated in this study. The chelating agents in the culture medium augmented with Cr(VI) improved the bioavailability of Cr in plants. In another study, EDTA application in Cr contaminated soil resulted in higher endogenous levels of Cr(III) in plants. Moreover, EDTA addition improved the growth by regulating Cr species, ion homeostasis and accumulation of secondary metabolites in castor bean (*Ricinus communis* L.) (Qureshi et al., 2020).

**Table 5:** Effect of chelates application for remediation of chromium in soil.

Plant species	Chelate applied	Concentration in biomass (mg kg^-1^)		(mg kg^-1^)	References
		Before	After		
Rice	25 µM EDTA	30	42	100	Huda et al. (2021)
Lemongrass	50 mg EDTA kg^-1^	12.2	17.93	50	Patra et al. (2018)
Alfalfa	0.14 g EDTA	2.45	4.10	50	Chigbo and Batty (2013)
Barnyard grass	10 mmol EDTA kg^-1^	79.50	109.23	79.50	Ebrahimi (2014)
Common reed (*Phragmites australis*)	10 mmol EDTA kg^-1^	0.002	125.71	550	Ebrahimi (2015)
Chinese mustard	2 mM EDTA kg^-1^	21	28	51.5	Han et al. (2004)
Mustard	10 mmol EDTA kg^-1^	1328	1411	169	Firdaus-E-Bareen and Tahira (2010)
Downy thorn apple	1 mmol EDTA kg^-1^	0.17	0.49	113	Jean et al. (2008)
Maize	7.5 mmol EDDS kg^-1^	0.003	0.019	151	Meers et al. (2008)
Rice	10 µM EDTA	0.0002	93.64		Mohanty and Patra (2012)
Wheat	10 µM DTPA	0.0003	110.25		Mohanty and Patra (2012)
Green gram	10 µM EDDHA	0.07	52.6		Mohanty and Patra (2012)
Water spinach	3 mg EDTA kg^-1^	400	7000	13217	Chen et al. (2010)
Physic nut	0.3 g EDTA kg^-1^	8	33	56.9	Jamil et al. (2009)
Sunflower	0.708 mM EDTA	2.98	4.88	30	January et al. (2008)
Sunflower	0.1 g EDTA kg^-1^	0.2	0.7	8.05	Turgut et al. (2004)
Sunflower	0.3 g EDTA kg^-1^	0.23	0.22	7.72	Turgut et al. (2005)

### 8.4 Remediation by nanoparticles

**Figure 4:**
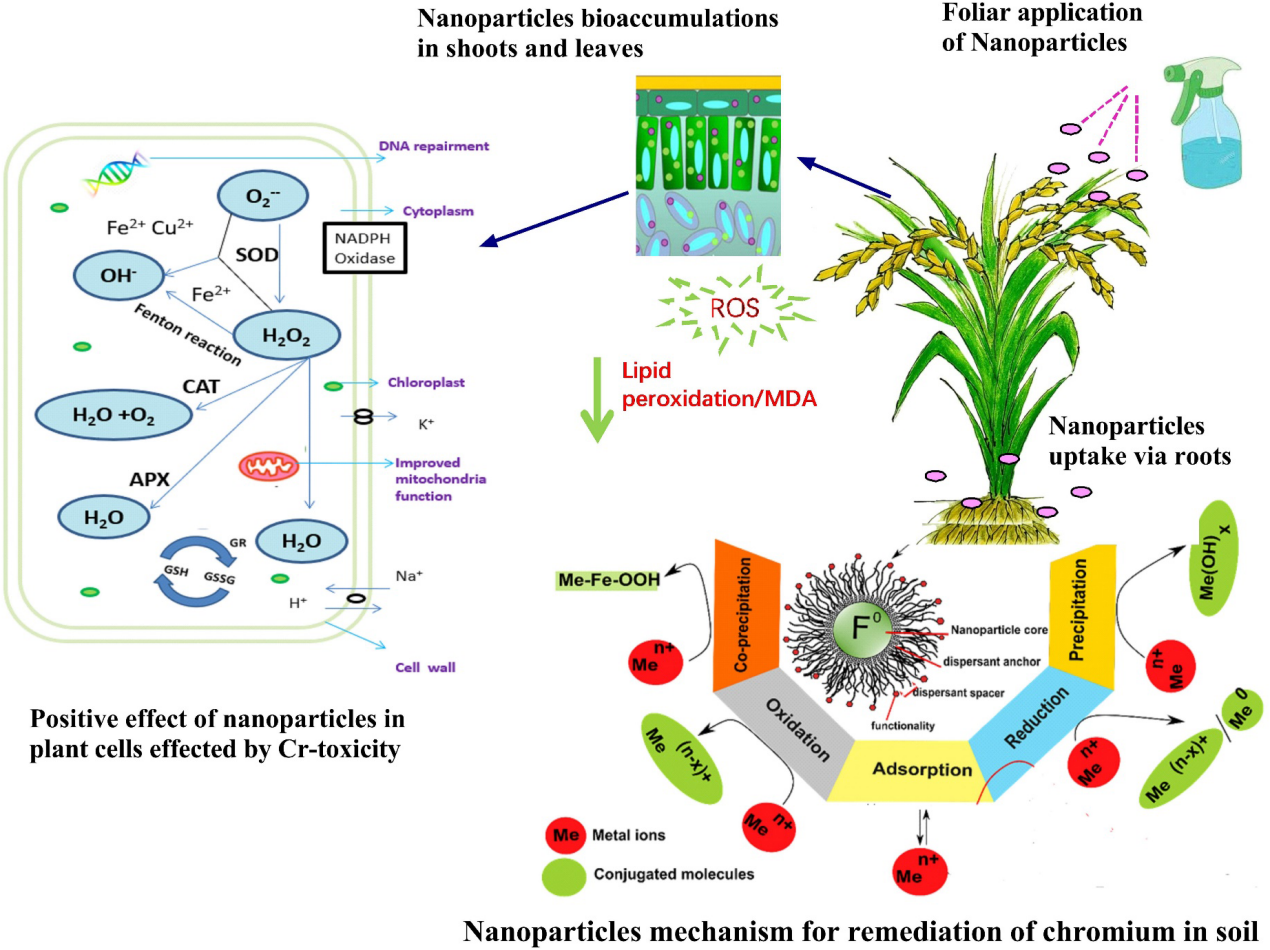
Nanoparticles (NPs) application reduced oxidative stress in plant species. Under chromium (Cr) toxicity, cellular respiration produces O_2_ **^.-^** that is converted into hydrogen peroxide by the activity of superoxide (SOD). H_2_O_2_ is then converted into O_2_ and H_2_O by the combined activities of ascorbate peroxidase (APX), catalase (CAT), glutathione reductase (GR), and glutathione peroxidase (GPX). NPs minimizes the accumulation of O_2_ **^.-^** and H_2_O_2_. The reactive oxygen species (ROS) which causes lipid peroxidation, enzyme inactivation, and cell death. The activity of ROS was significantly minimized by NPs due to improved production of antioxidants i.e., CAT, SOD, and POD (Ali et al., 2021). Mechanism representations i.e., precipitation, reduction, adsorption, oxidation, and coprecipitation for the decontamination of toxic trace-metals i.e., Cr in soil/aqueous medium by NPs with a core-shell structure (Yang et al., 2019).

Nano-remediation is an eco-friendly and cost-effective method of detoxifying heavy metals in soil and other environments using nanoparticles (NPs) (Ahmed et al., 2021a; Wei et al., 2022a; **Table 6**). By absorbing heavy metals, lowering the hazardous valence to a stable metallic state, and accelerating the reaction, this unique remediation strategy has been demonstrated to be efficient in the removal of toxic heavy metals (Mondal et al., 2020). Synthesis of nZVI NPs in colloidal solution using green tea extract having an average particle diameter of 5-10 nm with polyphenol coating (which served as a capping and reducing agent) was significantly effective in remediating Cr(VI) from groundwater passing through porous soil beds (Mystrioti et al., 2014). Synthesis of NPs by using various rose apple (*Syzgium jambos* L.), candlenut tree (*Aleurites moluccanus* L.), and oolong-tea leaves extracts were significantly remediate Cr(VI) from aqueous medium up to 90% at initial 5 minutes, due to its maximum NPs antioxidant property, but complete removal took after 60 minutes (Xiao et al., 2016). The removal effectiveness of Cr(VI) was greatly influenced by factors i.e., Cr(VI) initial concentration, NPs dosage, solution pH, and temperature. For a constant concentration of Cr, the availability of active sites rises with increasing NPs dosage, which improves the removal rate (Xiao Z. et al., 2017). Various probable processes for effective decontamination of inorganic pollutants by NPs have already been hypothesized throughout the application, including precipitation, adsorption, complexation, and reduction (Mondal et al., 2020; Ahmed et al., 2021b). The most well-known method for eliminating Cr is called as reduction, which is followed by adsorption. According to Li and Zhang (2007), whenever the trace-metal ions already had a greater negatively standard-redox strength (*E^0^*) as compared to, or were like Fe^0^ (-0.41 V), the method for decontamination of Cr *via* green-synthesized iron-NPs was largely regulated by surface complexation/adsorption. However, whenever the Cr ions already had substantially higher positive *E^0^* as compared to Fe^0^, precipitation and reduction of Cr ions predominate (Lin et al., 2019). When the Cr cations had somewhat more positive *E^0^* as compared to Fe^0^, both reduction and adsorption happened (Ahmed et al., 2021b). Other possibilities included co-precipitation and Fe-hydroxide oxidation (Sebastian et al., 2018; **Figure 4**). In addition, bimetallic Fe-NPs and Fe-oxide remove pollutants through catalytic degradation and adsorption respectively (Ahmed et al., 2021b). However, there are significant limitations and knowledge gaps that must be addressed to ensure social acceptance and safe usage of green synthesized NPs for toxic heavy metals remediation. As a result, more field studies are required to assess the application’s safety, reliability, efficacy, fate, intrinsic toxicity of NPs, and long-term impacts of NPs on Cr bioavailability and absorption in contaminated soils. To attain its promised implications in the environmental sector, future research should focus on doze optimization and safe targeted delivery of NPs.

**Table 6:** Application of nanoparticles for remediation of chromium in aqueous medium.

Initial conc. of Cr	NP source	NP type	Reaction time	Removal efficiency %	References
100 mg L^-1^	*Eucalyptus globulus*	nZVI	30 min	98.1%	Madhavi et al. (2013)
100 mg L^-1^	*Citrus maxima*	Fe-NPs	90 mins	99.29%	Wei et al. (2016)
15 mg L^-1^	*Eucalyptus globulus* leaves	nZVI	60 mins	58.9% Cr and 33.0% Cu	Weng et al. (2016)
50 mg L^-1^	*Syzygium jambos*, Oolong tea*, Aleurites moluccana*	Fe NPs	60 min	100%	Xiao et al. (2016)
300 mg L^-1^	*Rosa damascene*, *Thymus* *vulgaris*, and *Urtica dioica*	Fe-NPs	25 mins	100%	Fazlzadeh et al. (2017)
10 mg L^-1^	*Eucalyptus globulus* leaves	nZVI	35 mins	98.9%	Jin et al. (2017)
50 mg L^-1^	*Syzygium jambos* leaves	nZVI	90 mins	99.45%	Xiao Z. et al. (2017)
100 mg L^-1^	*Eichhornia crassipes* leaves	Fe-NPs	80 mins	89.9%	Wei et al. (2017)
100 mg L^-1^	*Eichhornia crassipes* leaves	nZVI	90 mins	89.9%	Wei et al. (2017)
40 g L^-1^	*Eucalyptus globulus*	Fe-NPs	12 hrs	98.6%.	Jin et al. (2017)

### 8.5 Use of organic amendments for remediation

Organic material is facilitated in soil deposition of Cr, according to Branzini and Zubillaga (2012). We postulated that soil comprising most of the humidified organic material had a lesser Cr accessibility, which would minimize Cr deposition in plants. The use of organic modifications in Cr polluted soils and their impact on reducing Cr absorption in plants have been reported in several studies.

#### 8.5.1 Biochar

Biochar is produced in a low oxygen atmosphere through the combustion of carbonaceous material collected from a range of sources (Tomczyk et al., 2020). Biochar has a higher porosity, extensive functional groups containing oxygen over its microscopic layer, and acts as an adsorbent to sequester heavy metals in soil (Xu and Fang, 2015). It has a larger surface area, a higher negative and stronger surface charge, biochar has higher absorption properties as compared to raw organic soil materials. Thus, biochar application enhances water holding capacity, reduce nutrient losses, and improve soil structure. In addition, biochar-containing soils have resemblance to organic pollutants (Yu et al., 2009; Haider et al., 2022a). Integrating biochar with other soil amendments before tillage activity, such as manure fertilizer, compost, or lime, will enhance sustainability by cutting down the amount of tillage practices needed (Haider et al., 2022b; Haider et al., 2022c). Biochar improves nutrient uptake by preventing their loss by leaching (Major, 2009). The incorporation of biochar minimizes the availability of Cr and its accumulation and toxicity in plants (**Table 7**). Muhammad et al. (2017) studied the use of wheat straw biochar significantly increased the yield of paddy rice, total organic carbon, and nitrogen and minimized nutrient leaching. Toxic metal concentrations like Cr Chinese cabbage (*Brassica rapa* subsp. pekinensis) (Xu W. et al., 2019), fenugreek (*Trigonella foenum-graecum*) (Raj et al., 2021), lettuce (*Lactuca sativa*) (Nigussie et al., 2012), radish (Nabavinia et al., 2015), maize (Abbas et al., 2020), rice (Khan S. et al., 2013), barley (Rajput et al., 2021), mustard (Choppala et al., 2015) were substantially reduced by biochar application. In recent days, application of nZVI iron nanoparticles loaded maize straw pyrolyzed biochar significantly effect to minimize the toxicity of Cr in aqueous medium (Wei et al., 2022b). However, pyrolysis temperature, type of feedstock soil type, and the influence of biochar on metal immobilization and assimilation vary within crops (Woldetsadik et al., 2016; Nkoh et al., 2022).

**Table 7:** Effects of biochar application on crops growth and Cr uptake, grown on Cr-contaminated soils.

Plant specie	Feedstock	Applied rate	Soil type	Exp. type	Cr	Effect of Cr	References
Lettuce	Maize stalk (500°C)	0, 5 and 10 t/ha	Clay	Pot	Cr	The biochar amendment resulted in a drop in Cr concentration or even an improvement in soil fertility and nutrient uptake.	Nigussie et al. (2012)
Maize	Sugarcane bagasse (350°C)	0%, 3%	Silty clay loam	Pot	Cr	Biochar application significantly improved the growth and antioxidant activity of maize with reduction in Cr accumulation	Bashir et al. (2021)
Maize	Sugarcane bagasse (500°C) and acidified manure	3% and 5%	Silty loam	Pot	Cr	The inclusion of sugarcane bagasse biochar has the power to mitigate Cr activity in polluted soil and accretion in maize plant roots and shoots.	Abbas et al. (2020)
Maize	Cow manure (420°C)	5 g kg^-1^		Pot	Cr	Biochar application caused greater liming effect, improved the plant growth and shoot/root ratio and enzymatic activities	Liu et al. (2020)
Mustard green	Rice husk and maple leaves (550°C)	0.5, 1 and 2% w/w	Loamy sand	Pot	Cu, Pb, Cr	Rice husk and maple leaves reduced both leaching and phytoavailability of metals	Nejad and Jung (2017)
Maize	Biochar (agriculture residues) (500°C)	0, 1, 2.5, 5 and 10% w/w	Loamy sand	Pot	Pb, Cr	Biochar helps in reduction of metals	Alaboudi et al. (2019)
	Solid waste compost, coal fly ash, and rice husk (300°C and 600°C)	2 and 5% w/w	Clay loam	Laboratory incubation study	Cr	Chromium toxicity reduced with the addition of biochar and soil amendment	Saffari et al. (2014)
Spring barley	Industrially obtained wood	2.5%	Metal polluted soil	Pot	Zn, Mn, Cr, Cd, Pb, Cu	The use of biochar combined with metal-tolerant bacteria efficiently remediate the soil contaminated with heavy metals	Rajput et al. (2021)
Rice	Sewage sludge (550°C)	5 and 10% w/w	loamy sand	Pot	Cr, Cu, Co	The incorporation of biochar to the soil boosted soil fertility while lowering hazardous metal bioaccumulation.	Khan S. et al. (2013)
Mustard	Chicken manure	0,50 g kg^-1^	Calcic red clay		Cr	In soil Cr(VI) transformed into Cr(III), decreased Cr in plants and boosted dry matter	Choppala et al. (2015)
Rice	Rice residues (straw, husk, bran) 500°C	5% w/w		Pot	Multi-metal contaminated soil	Metal uptake was slowed in rice seedlings, plant growth and biomass enhanced, and mineral content in iron plaque began to rise.	Zheng et al. (2012)
	Orchard prune residue (500°C)	0, 1, 5 and 10% w/w	clay		Mine tailings with Cr, Cu, Pb and Zn	Maximum utilization of biochar minimized leachable Cd, Pb, and Cr.	Fellet et al. (2011)
	Chitosan and Hematite (600°C)	1% w/w	Cr polluted calcareous soil	Plastic bag	Cr	Application of chitosan boosted Cr(VI) reduction from 28.53% (biochar) to 46.23% and inclusion of hematite from 28.55% (biochar) to 38.95%.	Zibaei et al. (2020)
	Poultry manure, cow manure, sheep manure biochar (450°C)	5% w/w		Incubation experiment	Cr	Biochar application helps in the reduction of Cr(VI) in contaminated soil	Mandal et al. (2017)
Maize and cowpea	Composted tannery sludge (CTS)	O, 2.5, 5,10, 20 Mg ha^-1^	Sandy loam	Field experiment	Cr	Due to the application of CTS and similar addition of Cr in roots and shoots leads to the higher growth of maize and cowpea plants	Sousa et al. (2018)
Cherry tomato (*Lycopersicon esculentum*)	Waste-water sludge (550°C)	10 t ha^-1^	Chromosol (Australian system)	Greenhouse pot trial	As, Cd, Cr, Cu, Pb, Zn	The application of biochar increased cherry tomato production by 64% and increased the availability of nutrients	Hossain et al. (2010)
Kidney vetch (*Anthyllis vulneraria*), Round-leaved Hellerkraut (*Noccaea rotundifolnum* L.), and alpine bluegrass (*Poa alpine* L.) alpine	Pruning residues from orchard (550°C), fir tree pellets and manure pellets mixed with fir tree pellets (350-400°C)	0, 1.5, and 3%	Technosol	Pot	Cd, cr	Different type of biochar promote plant growth for phytostabilization of mine tailing	Fellet et al. (2014)
Paddy rice	Whine lees (600°C)	0.5 and 1%		Pot	Cr, ni, cu, zn, cd, pb	Exchangeable Cr, Ni, Cu, Pb, Zn, and Cd decreased in soil due to increased soil pH and were also reduced in plant roots, stems, leaves, and rice husk with wine lees-derived BC.	Zhu et al. (2015)
Tomato (*Lycopersicon esculentum* L.)	Woody biomass (Gliricidia sepium) 900°C	1, 2.5 and 5% w/w	Serpentine soil	Pot	Ni, cr, mn	BC derived from woody biomass maximized the immobilization of Cr, Ni, and Mn in serpentine soil and minimized metal-induced toxicities in tomato plants.	Herath et al. (2015)

#### 8.5.2 Compost

Compost is a well decomposed organic material produced under anaerobic conditions (Stanislawska-Glubiak et al., 2015). Furthermore, supplying nutrients, the addition of organic composts in large amounts supplies nutrients and serves as a soil stabilizer to boost the soil physical properties. Organic composts have insignificant number of contaminants and metals and used in polluted soils to minimize the availability of metals (Park et al., 2011). Despite an increase in their overall content, vermicomposting most likely eliminates heavy metals by forming organic complexes. Vermicompost has greatly reduced the availability of metals to plants and is easily accessible at low costs and is thus known as a good replacement for minimizing the availability of the metal (Matos and Arruda, 2003). Additionally, the application of compost to achieve better crop quality in Cr-polluted soils is beneficial (**Table 8**). Besides, compost application to two ornamental plants lemon balm (*Melissa officinalis*) and begonia (*Begonia semperflorens*) reduced the accumulation of Cr in plant tissue (Rendina et al., 2011). Application of compost decreased the solubility of Cr in soil and rice plant assimilation. Moreover, h the addition of vermicompost significantly improved the growth and yield traits including chlorophyll contents, plant height, and number of tillers, straw yield, grain yield, and harvest index (Koka et al., 2019).

**Table 8:** Effect of organic amendments on remediation of chromium stress in different plant species.

Plant species	Soil type	Organic amendment	Applied dose	Cr effect	References
Maize	Heavy metal contaminated soil	Cow manure dust, poultry manure dust, vermicompost, barnyard grass dust	5 g kg^-1^	Immobilization from plant-originated organic material and phytoextraction from animal excreta helps to clean up heavy metal-contaminated dirt.	Naser et al. (2017)
	Sandy loam	Biosolid compost (sawdust and sewage sludge)	100 Mg ha^-1^	Solubility and mobility of Cr reduced with the application of biosolid compost.	Branzini and Zubillaga (2012)
Radish	Contaminated soil	Vermicompost, leaf compost, spent mushroom compost		Amendment of polluted soil with organic fertilizer negatively impacts Pb, Mn, Cr, and Cd availability, uptake, and translocation to radish.	Alam et al. (2020)
Fescue *(Festuca arundiacea)*	Silt loam	Composted cow manure	10% (by volume)	Organic amendment contaminates the soil from mobile Cr(VI) to immobile Cr(III).	Banks et al. (2006)
Chinese mustard	Fine sandy loam	Biosolid compost, fish manure, poultry manure and spent mushroom	100 g kg^-1^	Plant absorption of Cr solubility reduced with incorporation of organic amendment.	Bolan et al. (2003)
Lemon balm and begonia	Silt loam	Compost of cattle ruminal content and Sphagnum-moss peat	250-2000 mg kg^-1^	Supplication of organic amendment reduced the deposition of Cr in roots and shoots of plants and phytotoxic symptoms.	Rendina et al. (2011)
	Silt loam	Farmyard manure (FYM) and poultry manure	10% w/w	In polluted site, FYM diminished the incidence of metal toxicities.	Khan et al. (2012)
Dwarf beans	Technosol contaminated soil	Fresh ramial chipped wood and composted sewage sludge		Organic amendment minimized the heavy metal in contaminated soil.	Hattab et al. (2015)
Sunflower	Cr contaminated soil	Poultry manure and vermicompost	10 t ha^-1^ and 5 t ha^-1^	Incorporation of PM and VC reduced Cr(VI) to Cr(III), improved fertility and physical properties of Cr contaminated soil.	Sunitha and Mahimairaja (2014)
Cow pea (*Vigna unguiculata*)	Sandy soil	Composted tannery sludge	10 and 20 Mg ha^-1^	The level of Cr stabilizes in soil with the addition of organic matter.	Oliveira et al. (2015)
Barley and Maize	Fine sand	Compost	2% w/w	Application of compost converts the Cr(VI) to Cr(III) less toxic form.	Radziemska et al. (2019)
Rice		Farmyard manure and vermicompost	10 t ha^-1^ and 5 t ha^-1^	Application of FYM and VC attenuated the toxicity of Cr and prominently increased the growth, yield attributes and rice yield.	Koka et al. (2019)
	Heavy metal contaminated soil	Cow manure, sheep manure, sewage sludge, solid waste compost and biosolid compost		Organic and inorganic amendment reduced the toxicity of metals in soil and plants.	Gul et al. (2015)
Physic nut	Black cotton calcareous soil	Bio-sludge and bio-fertilizer		Major drop in metal assimilation by plant, when handled with bio-sludge and bio-fertilizer, which is linked to the retention of heavy metal(loid)s in soil	Juwarkar et al. (2008)
	Clay	Cattle dung compost, sugarcane dregs compost, rice bran and soybean meal	0.1% and 2% w/w	The increased decrease of Cr due to greater DOC and quickly degraded materials was linked to the organic amendment diminishing resin extractable Cr(VI) in soil.	Chiu et al. (2009)
Spinach		Poultry litter	3% and 5% w/w	Poultry litter potentially reduced the bioavailability of Cr in soil, significantly increased the chlorophyll contents of spinach.	Sehrish et al. (2019)
Maize		Mexican sunflower compost and cassava waste compost	0, 20 and 40 t ha^-1^	The concentration of heavy metal such as Cd, Cr, Zn, Cu, and Pb reduced with compost treatment at 40 t ha^-1^ dose.	Adejumo et al. (2011)
Potato (*Solanum tuberosum*)	Metal contaminated soil	Peat compost, vermicompost	10% w/w	Organic amendment increased starch yield, absolute dry substance, quantity and decreased reducing sugar in potatoes.	Angelova et al. (2010)
		Compost		Compost and microbial activity help to transform from Cr(VI) to Cr(III) form.	Shukla et al. (2009)
	Metal contaminated soil	Manure, compost, biosolid and municipal solid waste		Organic amendment enhanced bioremediation of metalloids and reduced the bioavailability of metals.	Park et al. (2011)

#### 8.5.3 Manures

Organic manures improve soil fertility and microbial productivity, leading to a substantial improvement in soil health. The influence of organic changes on metals(loids) functionality and bioavailability is determined by the strength of the organic matter, microbial population, and influence on chemical and physical properties of soil, or even the specific kind of soil and metals(loids) associated (Angelova et al., 2013). Farmyard manure (FYM) is the primary source of organic manure in the cropping system. FYM has a favorable impact on agricultural yields, enhancing the physical, chemical, and biological parameters of soil (Alam et al., 2014). The application of FYM in the soil to minimize Cr toxicity in Cr polluted soils for crop plants could be a useful approach (Singh et al., 2007). The preference for manure is a vital step in achieving the good efficacy of maize crop phytoextraction. Various organic manures, when applied to the soil, reduce the bioavailability and uptake of Cr (Naser et al., 2017). The rate of Cr reduction in soil was enhanced by organic amendments examined with mustard plants. Banks et al. (2006) studied the effect of growing plant and supplemental OM (cow manure) on Cr transported in soil. As organic matter level increases, chromate leaching decreased, followed by persistence on cation exchange sites or precipitation.

### 8.6 Genetic mechanisms to control Cr toxicity in plants

A significant problem is avoiding and reducing the harmful effects of heavy metals contamination in soil (Zeeshan et al., 2021). Genetic engineering can significantly improve a plant’s ability to transform, translocate, and lessen the adverse impacts of heavy metals (Raza et al., 2021). Omic tools have gained a lot of interest recently for their use in plant development and programs to mitigate agricultural production challenges, specially to mitigate heavy metal stress (Khan et al., 2021). To identify target genes, proteins, and metabolites linked to Cr detoxification and stress tolerance responses in plants, genomics, proteomics, and metabolomics have become effective methods (Chaudhary et al., 2019). It is possible to modify the Cr stress-responsive genes, proteins, and metabolites to either increase plant tolerance to Cr stress or decrease Cr accumulation (Thakur et al., 2019). Tools for genetic engineering that are particularly effective at changing the genes involved in the acquisition, transport, and accumulation of Cr inside the plant are necessary for this type of manipulation (Khan et al., 2021). The main goal of genetic engineering is the creation of tolerant varieties using either a transgenic approach or genome editing (Raza et al., 2021). Anwar and Kim (2020) reported that through genome editing active participation in the control of plant metabolism, essential genes important for increased metal tolerance have been developed into transgenics, which provide insights into how to understand and improve the tolerance capacity of plants. A successful method for creating resistant cultivars is to transfer candidate genes from plants with a high tendency for HM hyper-accumulation (Rahman et al., 2022).

The best way to reduce metal toxicity within cellular locations is to use transgenic plants with altered efficiencies for metal transport into vacuoles (Khan et al., 2021). Heavy metals (HM) transporter genes are thought to be potential candidates for genetic engineering to improve metal tolerance in plants (Zhang et al., 2018). OsMTP1 in cultivated tobacco (*Nicotiana tabacum*) and PgIREG1 in *Arabidopsis* are two examples of metal transporter genes that have been genetically modified (Merlot et al., 2014; Das et al., 2016). Other metal transporter genes include those that encode metal chelators, metallothioneins (MTs) (Peng et al., 2017), and genes associated with antioxidant machinery (Peng et al., 2017; Raza et al., 2021). The use of transgenic techniques to increase resistance to metal oxidation has also been documented. Transgenic hyperaccumulators may be created by manipulating the antioxidant system to maintain redox equilibrium to avoid the destruction of biomolecules such as DNA, proteins, and lipids and to maintain the structural and functional stability of cellular structures of plant under Cr stress (Du et al., 2019). Transgenic plants that overexpress antioxidant genes for SOD, CAT, and APX with reduced ROS generation under Cr stress have been created to prevent metal toxicity-induced oxidative stress (Gao et al., 2016). Additionally, enhanced antioxidant systems in transgenic lines are associated with higher growth performance in terms of photosynthesis, mineral uptake, maintenance of redox homeostasis, and enzyme activity (Khan et al., 2021). Although transgenic lines created for over-expression traits do not always show the expected benefits, they can nevertheless have positive consequences by influencing the alternative tolerance mechanisms.

The phytochelatins (PCs), which contain hazardous metal ions and are enzymatically generated from GSH, amino acids, organic acids, or MTs, are another crucial area for improving the Cr stress tolerance in plants (Yadav, 2020). It should be noted that only MTs have coding genes, but the production of other compounds (such as GSH, amino acids, and organic acids) is controlled by the actions of the enzymes involved. Better physiological and biochemical characteristics, including membrane function and antioxidant activity, are displayed by transformed plants (Khan et al., 2021). According to Ai et al. (2018), overexpression of MYB1 from grown radish improved PC and anthocyanin synthesis, giving transgenic Petunia higher resistance against several metal toxicities, including Cr. Improved growth and stomatal density were seen in MYB1 over-expressing lines mainly due to the maintenance of relative water content (RWC), chlorophyll, and antioxidant activity. Therefore, it can be concluded that transgenic research aimed at creating cultivars with improved metal tolerance will have a considerable impact on crop production in the future (Ai et al., 2018).

The engineering of transcription factors (TFs) that control the synthesis of important metabolic chemicals also has an impact on the Cr stress tolerance in addition to the previously described essential regulators of metal tolerance. Many TF gene families play a vital role in the ability of HMs to withstand stress, including R2R3-type MYB, ZAT6, Zinc-Finger type, bZIP, GeBP-LIKE 4 (GPL4), and NAC (Khan et al., 2021; Raza et al., 2021). It was noted that transgenic rice that overexpresses OsMYB-R1 has a noticeable increase in lateral roots, which was assumed to be related to improved tolerance to Cr (Tiwari et al., 2020). Further supporting the role of lateral roots in Cr tolerance is the correlation between the increase in lateral roots and a corresponding increase in auxin accumulation in transgenic lines as compared to wild type plants. Along with that, it was also thought that the OsMYB-R1 over-expressing lines had significantly higher antioxidant activity and proline accumulation, which were likely mediated by salicylic acid (SA) signaling and contributed to the transgenic rice’s ability to tolerate Cr (Tiwari et al., 2020). As a result, TFs are essential molecular regulators that help plants tolerate Cr stress and lessen the negative effects of exposure to metals, which supports plant growth and development. However, the identification and functional confirmation of several additional TFs from diverse TF families, many of which are still mostly unknown, could, therefore, be helpful in creating enhanced plant types with high HM tolerance.

## 9 Conclusion and future perspectives

This paper presents new perspectives on Cr toxicity in plants and provides a review of related research on Cr toxicity in the environment, mainly in water and soil. Cr exists primarily in three oxidative states: Cr (0), Cr(III), and Cr(VI) which are the most stable form of Cr. Cr (0) is the metallic kind, the kind of Cr(III), and Cr(VI) is the most preponderant in soil and water. The current review looked at the various negative impacts of Cr exposure in plants, both morphologically and physiologically. Cr can cause a variety of hazardous consequences in plants, including changes in the germination process and root, stem, and leaf growth, as well as detrimental impacts on morphological and physiological systems like photosynthesis, water relations, and mineral nutrition. The hazardous qualities of Cr(VI) stem from its action as an oxidizing agent and the generation of free radicals during the reduction of Cr(VI) to Cr(III) that happens within the cell. Apart from generating reactive oxygen species (ROS), Cr(III) in the contrary can induce hazardous effects when present in large amounts because of its propensity to coordinate diverse chemical molecules, resulting in inhibition of metalloenzyme systems. Several approaches for viable alleviation of Cr-induced phytotoxicity have been used to combat this threat. Bioremediation, which involves phytoremediation (phytoextraction, phytodegradation, phytovolatilization, rhizosphere destruction, rhizofiltration, phytostabilization, and phytorestoration), and microbial treatment are the most common solutions (bacteria and fungi). Exogenous use of chelates, organic amendments (biochar, manure, and compost), and nano-remediation supplements are some more current Cr decontamination approaches. The findings of this review support the development of innovative and useful methods to limit the bioavailability and toxicity of chromium and the sustainable management of chromium-contaminated soil/water, thus benefiting the environment and public health. Harmful threats must be mitigated.

Chromium contamination in soil continues to increase with the increase in global production and use of the metal, which could endanger the lives of animals, plants, and humans. To better understand the ecological harm caused by Cr and practical remediation methods, this study concentrates on the biogeochemical behavior of Cr in soil-plant systems and the application of organic and inorganic amendments to reclaim Cr(VI)-contaminated soils. According to recent studies, there are significant differences in various chemical forms of Cr in terms of its solubility, mobility, adsorption/desorption, toxicity, bioavailability, and transformation. Chromium uptake and transport in soil plant systems is largely influenced by soil physicochemical characteristics (soil pH, EC, CEC, OM, manganese and iron oxides, microorganisms, etc.). When Cr enters plant cells through the pathways of necessary nutrients like Fe, sulphate, and phosphate, it might result in physiological and molecular alterations. Cr buildup affects nutrient intake, photosynthesis, growth, and development, and seed germination. High Cr concentrations can cause oxidative stress in plants and alter the structure of cell nuclei and chloroplasts. Overproduction of ROS could disrupt cell homoeostasis, stop cell division, harm DNA, and even cause cell death. Organic and inorganic reductants have been widely employed for the *in-situ* remediation of Cr(VI)-contaminated soil to lessen the hazard of Cr(VI) to soil-plant systems. Chemical, physical, and microbiological methods, as well as phytoremediation, have all been developed as countermeasures for Cr polluted soil cleanup over the previous few decades. It is especially helpful to use microorganisms to eliminate Cr from the environment. Numerous advantages of microbial remediation include lower costs and more public acceptance. Phytoremediation is a useful alternative that does away with the requirement for moving and excavating soil. However, compared to the total area of contamination, the area completely decontaminated by bacteria, and phytoremediation is substantially smaller. This study demonstrates that many environmental Cr-related concerns remain poorly understood even though several studies have been done in recent years. These include the distribution patterns of Cr in plants, the soil-plant uptake of Cr, the geochemical behavior of Cr in soil, and the process of Cr buildup. Furthermore, there is still disagreement regarding the potential environmental risks associated with the use of organic and inorganic reductants for the remediation of Cr(VI)-contaminated soils. This is because there is little knowledge about these risks. Therefore, the need for new Cr-pollution reduction strategies is urgent. With improvements in our understanding of the reciprocal interactions between the immune and neurological systems, the microbiome is increasingly seen as a crucial component of both human and animal health. We must be knowledgeable about the numerous chemical, physical, and biological remediation techniques and their corresponding benefits and drawbacks if we are to successfully combat the global threat of Cr pollution and toxicity. More research is needed to understand localization and partitioning of chromium in plant cells, determination of ROS producing and scavenging pathways, and analyzing how Ca^2+^ homeostasis regulates these interactions to elucidate complete Cr metabolic and detoxification mechanisms. Current research focuses on the efficiency of reduction and stabilization of reducing agents, but very little attention has been paid to the long-term stability of reduced Cr(III) in amended soils. Due to the complexity and diversity of soil systems, immobilized Cr(III) can be re-oxidized to Cr(VI) and remobilized. Therefore, it is necessary to investigate the long-term stability of chromium (III) in amended soils. Since reducing agents, especially nanomaterials can affect physical and chemical properties of soil, their potential impact on soil properties and biodiversity should also be considered to assess their ecological risks. Nevertheless, as we continue to grasp the molecular processes underlying Cr toxicity, we will be able to develop novel, more potent treatment approaches to reverse the harm exposure to this metal causes to human health. The review’s observations should aid in the development of creative and useful methods for limiting Cr bioavailability and toxicity and sustainably managing Cr-polluted soils/water, hence reducing its dangers to the environment and public health.

## Author contributions

UZ: Conceptualization, Data collection and analysis, Writing – original draft. MA: Validation and Formal analysis. MM: Visualization, Formal analysis. SH: Writing – review and editing, Methodology and Supervision. BS: Contribution to study design, Software. BA: Visualization, Formal analysis. MI, SA and IK: Software, Formal analyses. FH, MW, MT and SE: Writing–review and editing, and Resource. All authors contributed to the article and approved the submitted version.
